# Activation of the Vpdm^VGLUT1^-VPM pathway contributes to anxiety-like behaviors induced by malocclusion

**DOI:** 10.3389/fncel.2022.995345

**Published:** 2022-12-20

**Authors:** Yuan-Yuan Ji, Xin Liu, Xin Li, Yi-Fan Xiao, Teng Ma, Jian Wang, Yue Feng, Juan Shi, Mei-Qing Wang, Jin-Lian Li, Jiang-Hua Lai

**Affiliations:** ^1^College of Forensic Science, Xi’an Jiaotong University, Xi’an, China; ^2^Department of Anatomy, School of Medicine, Northwest University, Xi’an, China; ^3^Department of Anatomy, K. K. Leung Brain Research Centre, Fourth Military Medical University, Xi’an, China; ^4^State Key Laboratory of Military Stomatology, Department of Oral Anatomy and Physiology, National Clinical Research Center for Oral Diseases, Shaanxi International Joint Research Center for Oral Diseases, School of Stomatology, Fourth Military Medical University, Xi’an, China; ^5^Department of Stomatology, The 960th Hospital of People’s Liberation Army, Jinan, China; ^6^Functional and Molecular Imaging Key Lab of Shaanxi Province, Department of Radiology, Tangdu Hospital, Fourth Military Medical University, Xi’an, China; ^7^Department of Neurosurgery, Tangdu Hospital, Fourth Military Medical University, Xi’an, China

**Keywords:** principal sensory trigeminal nucleus, ventral posteromedial nucleus of thalamus, unilateral anterior crossbite, temporomandibular disorder, vesicular glutamate transporter 1, anxiety

## Abstract

Occlusal disharmony has a negative impact on emotion. The mesencephalic trigeminal nucleus (Vme) neurons are the primary afferent nuclei that convey proprioceptive information from proprioceptors and low-threshold mechanoreceptors in the periodontal ligament and jaw muscles in the cranio-oro-facial regions. The dorsomedial part of the principal sensory trigeminal nucleus (Vpdm) and the ventral posteromedial nucleus (VPM) of thalamus have been proven to be crucial relay stations in ascending pathway of proprioception. The VPM sends numerous projections to primary somatosensory areas (SI), which modulate emotion processing. The present study aimed to demonstrate the ascending trigeminal-thalamic-cortex pathway which would mediate malocclusion-induced negative emotion. Unilateral anterior crossbite (UAC) model created by disturbing the dental occlusion was applied. Tract-tracing techniques were used to identify the existence of Vme-Vpdm-VPM pathway and Vpdm-VPM-SI pathway. Chemogenetic and optogenetic methods were taken to modulate the activation of Vpdm^VGLUT1^ neurons and the Vpdm-VPM pathway. Morphological evidence indicated the involvement of the Vme-Vpdm-VPM pathway, Vpdm-VPM-SI pathway and Vpdm^VGLUT1^-VPM pathway in orofacial proprioception in wild-type mice and vesicular glutamate transporter 1 (^VGLUT1^): tdTomato mice, respectively. Furthermore, chemogenetic inhibition of Vpdm^VGLUT1^ neurons and the Vpdm-VPM pathway alleviated anxiety-like behaviors in a unilateral anterior crossbite (UAC) model, whereas chemogenetic activation induced anxiety-like behaviors in controls and did not aggravate these behaviors in UAC mice. Finally, optogenetic inhibition of the Vpdm^VGLUT1^-VPM pathway in VGLUT1-IRES-Cre mice reversed UAC-induced anxiety comorbidity. In conclusion, these results suggest that the Vpdm^VGLUT1^-VPM neural pathway participates in the modulation of malocclusion-induced anxiety comorbidity. These findings provide new insights into the links between occlusion and emotion and deepen our understanding of the impact of occlusal disharmony on brain dysfunction.

## Introduction

Temporomandibular disorders (TMDs) include functional disorders affecting the stomatognathic system, especially the masticatory muscles, temporomandibular joint (TMJ) and related structures. These disorders are characterized by joint and/or muscular pain, joint noise, limited mandibular function and negative emotions (Armijo-Olivo et al., 2016). The etiology of TMD is controversial in clinical dentistry (Bonjardim et al., 2005). Some potential factors that increase the disease risk are occlusal conditions, parafunctional habits, muscular hyperactivity, postural characteristics, and psychosocial factors, such as anxiety, depression and socioeconomic conditions (Melo et al., 2020). According to previous animal experiments (Henderson et al., 2015; Zhang et al., 2016) and cross-sectional studies (Wang et al., 2009), aberrant dental occlusions negatively impact TMDs. Occlusions regulate muscular activity via periodontal-muscular feedback mechanisms that are adjusted by contact-mediated loading (Wang and Mehta, 2013). Contact changes, such as posterior crossbite, negatively affect normal chewing movements in humans (Piancino et al., 2006, 2012). In animal studies, artificial appliances were inserted and composite resin was deposited on the molars to induce degenerative responses in the mandibular condylar cartilage in the TMJ (Jung et al., 2014; Henderson et al., 2015; Wu et al., 2019). Moreover, a unilateral anterior crossbite (UAC) animal model that induced osteoarthritic lesions in the TMJ was developed (Zhang J. et al., 2018). In previous studies, we observed that UAC elicited hyperactivity in the jaw closing muscles, most likely by activating the neural pathway of the periodontal afferents of the mesencephalic trigeminal nucleus (Vme) to trigeminal motor neurons (Liu et al., 2017).

Previous research on animals and humans has demonstrated that sensory information from the oral cavity is transmitted to the central nervous system (Ono et al., 2010). Moreover, occlusion and cognitive function are correlated. Normal occlusion helps to maintain cognitive function in various brain regions, including the hippocampus, a brain area that is crucial for learning and memory (Teixeira et al., 2014). Reduced mastication induced by occlusal disharmony in animals acts as chronic stress and induces functional and morphological changes in the hippocampus, thus suppressing spatial learning and memory functions (Ono et al., 2010; Alvarenga et al., 2019). Data from a human study indicated an association between tooth loss and the prevalence of dementia in participants, implying that reduced mastication may be a risk factor for Alzheimer’s disease (Stein et al., 2007). In addition, malocclusion has been shown to induce anxiety-like behaviors (Liu et al., 2019) and aggravate anxiety levels in psychologically stressed rats, which manifested as notable changes in behaviors and the expression of associated transmitters and receptors (Tang et al., 2017). Occlusal splint treatments, which are widely used in clinical settings and restore neuromuscular balance by balancing occlusal contacts, have been reported to positively influence anxiety-related symptoms (Melo et al., 2020). These results imply that occlusion may impact emotion modulation.

Vme neurons are the primary afferents that convey proprioception of the periodontal ligament and jaw muscles to the central nervous system (Liu et al., 2017). The dorsomedial part of the principal sensory trigeminal nucleus (Vpdm) is an important Vme projection target and serves different functions, including relaying and processing trigeminal proprioceptive information (Luo et al., 1991). The trigeminal sensory nuclear complex, including the Vpdm and the spinal trigeminal nucleus, is responsible for transmitting cranio-oro-facial neural signals to the thalamus, mainly to the ventral posteromedial nucleus (VPM), before the signals are sent to the somatosensory cortex for perception (Dong and Dong, 2018). Vme neurons send afferent signals to VPM-projecting neurons in the Vpdm (Luo and Dessem, 1995). Furthermore, the primary somatosensory areas (SI) have been reported to receive thalamic inputs mainly from the ventrobasal complex of the thalamus, which includes the ventroposterolateral nucleus and VPM (Dong and Dong, 2018). Numerous studies have shown that the somatosensory cortex is involved in every stage of emotional processing, including recognizing emotional changes in stimuli, generating emotional states, and regulating emotions (Kropf et al., 2019). The SI is also extensively connected to the limbic system, which plays a critical role in mediating emotion disorders (Price, 2000; Ossipov et al., 2010). Moreover, optical inhibition of CaMKII-positive neurons in the SI has been shown to significantly alleviate anxiety-like behaviors (Jin et al., 2020).

Vesicular glutamate transporters (VGLUTs) are responsible for loading glutamate into synaptic vesicles (Bai et al., 2001). Variations in VGLUT molecule expression can reflect changes in synaptic transmission (Wojcik et al., 2004). VGLUT1 and VGLUT2 have been identified as special markers for glutamatergic neurons. Moreover, VGLUT1 and VGLUT2 have been shown to have complementary distributions in various brain regions (Kaneko and Fujiyama, 2002; Ge et al., 2010; Zhang F. X. et al., 2018). Unlike their complementary distribution in other brain areas, many neurons in the Vpdm and VPM coexpress both VGLUT1 and VGLUT2 (Nakamura et al., 2005; Barroso-Chinea et al., 2008; Graziano et al., 2008; Ge et al., 2010). Further the projections from the VPM to the SI display VGLUT1 and VGLUT2 immunostaining, which is consistent with the colocalization of VGLUTs at the mRNA level in the VPM (Nakamura et al., 2005).

Our previous studies indicated that UAC stimulated the expression of VGLUT1 in the Vme and Vpdm (Liu et al., 2017; Shi et al., 2021). Thus, we aimed to determine whether there exists a putative Vme-Vpdm-VPM-SI glutamatergic neural pathway that dominates the emergence and regulation of anxiety-like behaviors stimulated by aberrant dental biomechanical stress. To test this hypothesis, optogenetics, chemogenetics, morphology, and behavioral approaches were applied to prove that this neural pathway is involved in malocclusion-induced negative emotions. In the present study, we used morphological approaches to establish the Vme-Vpdm-VPM-SI neural pathway. Chemogenetics was also carried out to demonstrate the involvement of the Vpdm-VPM neural pathway in malocclusion-related anxiety-like behaviors. Moreover, we applied optogenetic method to further prove the anxiety modulating role that the VGLUT1-expressing Vpdm neurons might play in this neural pathway.

## Materials and methods

### Animals

The following mouse lines (8–12 weeks, male, 20–30 g) were used in this study: C57BL/6J, VGLUT1-IRES-Cre and Ai9 Cre reporter mice. VGLUT1:tdTomato: VGLUT1-IRES-Cre mice were crossed with Ai9 mice. All mice were purchased from the Laboratory Animal Central of the Fourth Military Medical University or Jackson Laboratory and housed in a temperature- (22–25°C) and humidity-controlled (50% ± 10%) environment with a 12 h light/dark cycle. All experimental protocols were performed in accordance with the Animal Care and Use Committees of The Fourth Military Medical University (Xi’an, China).

The present study consisted of five parts. The first part aimed to determine whether UAC induces anxiety-like behaviors at different time points. Mice were randomly divided into a control (*n* = 7, 1 week; *n* = 7, 2 weeks; *n* = 7, 4 weeks; *n* = 8, 6 weeks) and UAC group (*n* = 8, 1 week; *n* = 7, 2 weeks; *n* = 8, 4 weeks; *n* = 7, 6 weeks). The second part involved detecting whether Vpdm^VGLUT1^ neurons play a sufficient and necessary role in UAC-induced anxiety comorbidity. In the hM4Di-inhibiting Vpdm^VGLUT1^ neurons experiment, mice were studied under mCherry-CNO (Control: *n* = 6; UAC: *n* = 6), hM4Di-SAL (Control: *n* = 8; UAC: *n* = 8) and hM4Di-CNO (Control: *n* = 7; UAC: *n* = 8) manipulation. In the hM3Dq-activating Vpdm^VGLUT1^ neurons experiment, the mice were randomly divided into hM3Dq-SAL-treated control mice (*n* = 7), hM3Dq-SAL-treated UAC mice (*n* = 8), hM3Dq-CNO-treated control mice (*n* = 8) and hM3Dq-CNO-treated UAC mice (*n* = 7). The third part aimed to confirm the Vme-Vpdm-VPM and Vpdm-VPM-SI pathways and Vpdm^VGLUT1^ neurons participating in UAC-induced anxiety comorbidity. Three to four mice were adopted for morphological detection and c-FOS counting. The fourth part aimed to investigate whether the Vpdm-VPM pathway plays a sufficient and necessary role in comorbid anxiety. In the hM4Di-inhibiting experiment, mice were under mCherry-CNO (Control: *n* = 7; UAC: *n* = 6), hM4Di-SAL (Control: *n* = 7; UAC: *n* = 8) and hM4Di-CNO (Control: *n* = 8; UAC: *n* = 8) manipulation. In the hM3Dq-activating experiment, mice were randomly divided into hM3Dq-SAL-treated control mice (*n* = 8), hM3Dq-SAL-treated UAC mice (*n* = 8), hM3Dq-CNO-treated control mice (*n* = 6) and hM3Dq-CNO-treated UAC mice (*n* = 8). The fifth part aimed to determine whether the Vpdm^VGLUT1^-VPM pathway participates in the modulation of comorbid anxiety. In the UAC group, mice were under mCherry (*n* = 7) or eNpHR (*n* = 7) manipulation. In the control group, mice were under mCherry (*n* = 6) or eNpHR (*n* = 7) manipulation.

### UAC modeling

As we previously described (Liu et al., 2017), UAC operations were performed on 8-week-old mice. Mice were anesthetized with 40 mg/kg pentobarbital sodium. Each mouse has two pairs of incisors, and under normal conditions, the maxillary incisors bite on the labial side of the mandibular incisors. The UAC model was established by fixing metal tubes made of a pinhead (inner diameter = 0.61 mm; thickness = 0.3 mm; the maxillary tube was 1.5 mm long and the mandibular tube was 2.5 mm long) onto the left maxillary and mandibular incisors. The mandibular tube was bent to form a 135° labially inclined occlusal plate to create a crossbite relation with the maxillary-tubed incisor (Supplementary Figure 1A). Normal mandibular movement was interrupted by the conflicting guidance of the untubed right incisors with a normal overjet relation and the tubed left incisors with a crossbite relation. The tubes were carefully bonded with zinc phosphate cement and checked every 2 days. We ensured that the placement of the metal tubing remained constant during the experimental period. The mice in the control group received the same procedure except the metal tube bonding. All animals received the same diet and were fed cylindrically shaped pressed food pellets. Based on a previous study (Jing et al., 2017), short-term orofacial hyperalgesia instead of chronic pain was induced by UAC. Compared with the control group, the weight of the UAC model decreased on Day 1 but recovered by Day 3. There was no significant difference in weight between the UAC and control groups after the 7th day (Jing et al., 2017).

### Open field test and elevated plus maze

To examine the effect of UAC on anxiety in mice, the open field test (OFT) and elevated plus maze (EPM) test were conducted to assess anxiety-like behaviors (Yang et al., 2018; Yin et al., 2020). The OFT was conducted to evaluate anxious emotions and general locomotor abilities. A white square arena (50 × 50 × 45 cm) under homogenous illumination was used in the OFT. The arena was divided into a central area and a peripheral area (Hölter et al., 2015). Each mouse was placed in any corner of the open field and allowed to explore for 15 min. The total distance traveled, time spent in the central area and distance traveled in the central area were recorded by an automated analysis system. All animals were habituated in the behavior testing room for 30 min before the behavioral tests, and experimenters were blinded to the group allocation and outcome assessment.

During the EPM, mice explored a plus shaped maze (30 cm × 5 cm). Two arms were closed (wall height: 25 cm), and two arms were open. Each mouse was placed in the central square of the maze and allowed to freely explore for 5 min. The number of entries and the time spent in the open arms were calculated.

### Stereotaxic surgery

The anesthetized mice were fixed in a stereotaxic frame (Narishige Scientific Instrument Lab, Tokyo, Japan) after the pain withdrawal reflex disappeared and were injected with tracers or viruses through a glass micropipette attached to a 1-μl Hamilton microsyringe. All tracers and viruses were injected at a rate of 30 nl/min using a syringe pump. All viruses were purchased from BrainVTA (BrainVTA, Wuhan, China).

For chemogenetic inhibition or activation of VGLUT1-ir neurons in the Vpdm, VGLUT1-IRES-Cre mice were bilaterally injected with AAV2/9-Ef1α-DIO-hM4D(Gi)-mCherry-WPREs (200 nl, 5.18 × 10^12^ vg/mL), AAV2/9-Ef1α-DIO-hM3D(Gq)-mCherry-WPREs (200 nl, 5.27 × 10^12^ vg/mL) or AAV2/9-Ef1α-DIO-mCherry-WPRE-hGH pA (200 nl, 5.18 × 10^12^ vg/mL) in the Vpdm [coordinates relative to Bregma: anterior–posterior (AP): –5.30 mm; medial–lateral (ML): ± 2.00 mm; dorsal–ventral (DV):−4.30 mm].

For the tracing experiment, wild-type mice were injected unilaterally with 200 nl biotinylated dextran amine (BDA; D1956, 10,000 MW; Invitrogen, Carlsbad, CA, USA) into the Vme (AP: –0.95 mm, ML: 5.52 mm, DV: –3.60 mm) or Vpdm (AP: –5.30 mm, ML: 2.00 mm, DV: –4.30 mm) and also injected with 40 nl 4% Fluoro-Gold (FG; 80014, Biotium, CA, USA) into the contralateral VPM (AP: –1.70 mm, ML: –1.50 mm, DV: –3.70 mm) or SI (AP: + 1.30 mm, ML: –2.60 mm, DV: –2.50 mm). Alternatively, FG (40 nl) was injected unilaterally into the Vpdm of C57BL/6J or the VPM of VGLUT1:tdTomato mice, and AAV2/9-hSyn-DIO-Synaptophysin-mCherry-WPRE-hGH pA (200 nl, 2.45 × 10^12^ vg/mL) was injected into the Vpdm of VGLUT1-IRES-Cre mice.

For chemogenetic inhibition or activation of Vpdm-VPM-projecting neurons, wild-type mice were bilaterally injected with AAV2/R-hSyn-CRE-WPRE-hGH pA (200 nl, 5.22 × 10^12^ vg/mL) into the VPM (AP: –1.70 mm, ML: ± 1.50 mm, DV: –3.70 mm) and AAV2/9-CaMKIIa-DIO-hM4D(Gi)-mCherry-WPRE-hGH pA (200 nl, 5.33 × 10^12^ vg/mL), AAV2/9-CaMKIIa -DIO-hM3D(Gq)-mCherry-WPRE-hGH pA (200 nl, 2.81 × 10^12^ vg/mL) or AAV2/9-CaMKIIa-DIO-mCherry-WPRE-hGH pA (200 nl, 6.36 × 10^12^ vg/mL) into the Vpdm.

For optogenetic inhibition of the Vpdm^VGLUT1^-VPM pathway, VGLUT1-IRES-Cre mice were bilaterally injected with AAV2/9-Ef1α-DIO-eNpHR3.0-mCherry-WPRE-hGH-pA (200 nl, 6.50 × 10^12^ vg/mL) or AAV2/9-Ef1α-DIO-mCherry-WPRE-hGH pA (200 nl, 5.18 × 10^12^ vg/mL) into the Vpdm. After 1 week, the animals were bilaterally implanted with optical fibers [200 μm in diameter, 0.37 numerical aperture (NA), Newdoon, Hangzhou, China] 200 μm above the VPM (AP: –1.70 mm, ML: ± 1.50 mm, DV: –3.50 mm). All mice were allowed to recover for 2 weeks and then tested for anxiety-like behaviors under yellow light stimulation.

### Optogenetic experiments

An optical fiber was attached to a fiber-coupled yellow (590 nm) laser (Newdoon). For the OFT, behavior was recorded during three 5-min periods: a prestimulation light-off period, a light-on period, and a poststimulation light-off period. For the EPM, the 9-min session was divided into three 3-min sections (light off-on-off sections). During the light-on period, yellow light was delivered continuously at 20 Hz (5 mW).

### Immunofluorescence histochemical staining

The immunofluorescence histochemical experiments were performed as described in our previous study (Ge et al., 2010; Hölter et al., 2015). Mice were perfused with 0.01 mol/L phosphate-buffered saline (PBS, pH = 7.4) and 200 ml 0.1 M phosphate buffer (PB, pH = 7.4) containing 4% (w/v) paraformaldehyde. The brains were obtained and immersed in 0.1 M PB containing 30% (w/v) sucrose overnight at 4°C. Transverse brain sections (25 μm) were cut with a cryostat (Leica CM1800; Heidelberg, Germany). The sections were rinsed in 0.01 M PBS and blocked with 10% normal donkey serum in 0.01 M PBS for 30 min at room temperature (RT). For c-FOS/mCherry/DAPI immunofluorescence histochemical staining, the sections were incubated overnight at RT with primary antibodies: mouse anti-c-FOS (1:400; ab11959, Abcam, Cambridge, MA, USA) antibody. Next, the sections were washed and incubated for 6 h at RT with Alexa 488-conjugated donkey anti-mouse IgG (1:500; R37114, Invitrogen). Finally, the nuclei were stained with DAPI (1:500; sc-3598, Santa Cruz, Dallas, TX, USA) for 10 min. For BDA/FG immunofluorescence histochemical staining, the sections were incubated with rabbit anti-FG (1:1000; ab153, Merck Millipore, Darmstadt, Germany) antibody overnight at RT. Next, the sections were incubated with a mixture of Alexa 647-conjugated donkey anti-rabbit IgG (1:500; A31573, Invitrogen) and Alexa 594-conjugated streptavidin (1:500; S32356, Invitrogen) or Alexa 594-conjugated donkey anti-rabbit IgG (1:500; A21207, Invitrogen) and Alexa 488-conjugated streptavidin (1:500; S11223, Invitrogen) antibody for 6 h at RT. For c-FOS/tdTomato/FG immunofluorescence histochemical staining, the sections were incubated with mouse anti-c-FOS and rabbit anti-FG antibodies overnight at RT. Next, the sections were incubated with Alexa 488-conjugated donkey anti-mouse IgG and Alexa 647-conjugated donkey anti-rabbit IgG antibodies for 6 h at RT. For VGLUT1/NeuN/mCherry immunofluorescence histochemical staining, the sections were incubated with rabbit anti-VGLUT1 (1:200; 135302, Synaptic Systems, Goettingen, Germany) and mouse anti-NeuN (1:400; MAB377, Merck Millipore) antibodies overnight at RT. Next, the sections were incubated with Alexa 488-conjugated donkey anti-rabbit IgG (1:500; R37118, Invitrogen) and Alexa 647-conjugated donkey anti-mouse IgG (1:500; A31571, Invitrogen) antibodies for 6 h at RT. Finally, the sections were mounted on glass slides, and confocal images were obtained with a laser scanning confocal microscope (Olympus) equipped with FV1000 (Ver. 1.7a) software. The image size at 10× and 20× magnification was 1024 × 1024 (pixel). The images at 60× and 180× magnification were obtained by z-stacks from many different z-depth images (z dimension: 6–11 slices, 1.0–3.0 μm/slice). Especially in the tracing experiments, in the Figures 4D1–D3 (bottom), the images were superimposed from 11 slices (2.0 μm/slice). In the Figures 4H1–H3 (bottom), the images were superimposed from 6 slices (1.0 μm/slice). In the Figures 4J, K (bottom), the images were superimposed from 11 slices (2.0 μm/slice). In the Figures 4N1–N4 (bottom), the images were superimposed from 6 slices (3.0 μm/slice). Then, we used FV1000 to modify the fluorescence intensity in different channel. Tiled images (low magnification) from the same brain slice were stitched into a single image containing the target nucleus.

**FIGURE 1:**
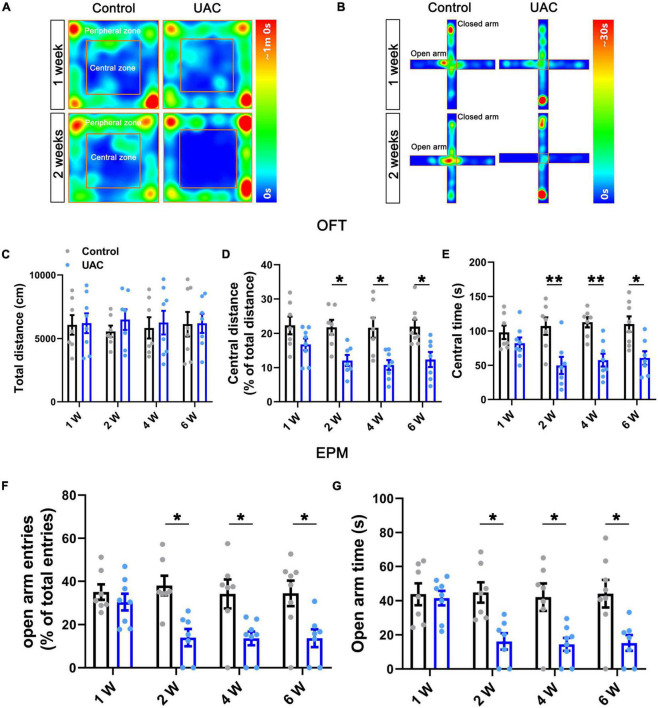
Mice with UAC showed anxiety-like behaviors. Heatmap during the OFT **(A)** and EPM **(B)** showing the effects of UAC in mice. Top: 1 week after UAC; bottom: 2 weeks after UAC. General locomotor activities was assessed by the total distance traveled **(C)** and anxiety-like behaviors were assessed by the percentage of central distance (distance traveled in the central area versus the total distance traveled) **(D)** and the time spent in central area **(E)** in the OFT and the percentage of open arm entries (open arms entries versus total entries) **(F)** and the time spent in the open arms **(G)** in the EPM after 1 week (Control: *n* = 7; UAC: *n* = 8), 2 weeks (Control: *n* = 7; UAC: *n* = 7), 4 weeks (Control: *n* = 7; UAC: *n* = 8) and 6 (Control: *n* = 8; UAC: *n* = 7) weeks of UAC. Data are presented as the mean ± SEM. *P < 0.05, **P < 0.01, compared with the control group.

**FIGURE 2:**
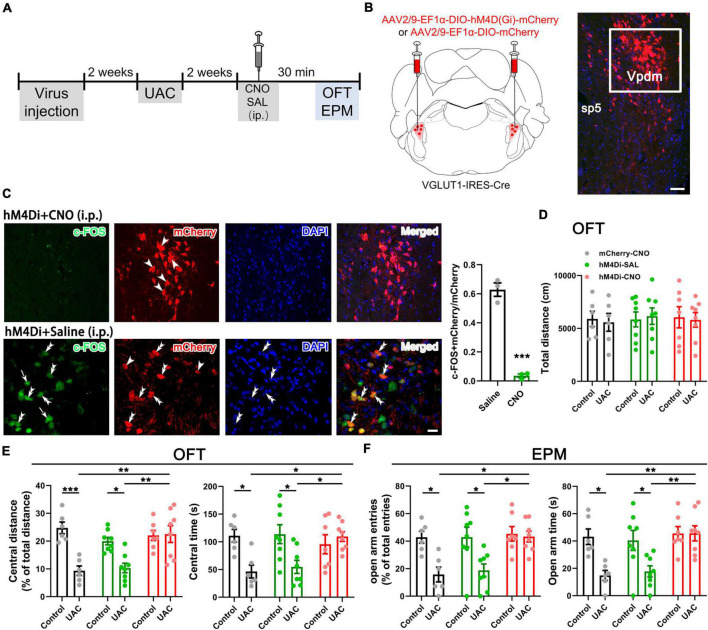
Chemogenetic inhibition of VGLUT1-ir neurons in the Vpdm alleviates UAC-induced anxiety-like behaviors. **(A)** Timeline of chemogenetic inhibition of Vpdm^VGLUT1^ neurons to investigate anxiety comorbidity. **(B)** Schematic showing the virus injection into the Vpdm of VGLUT1-IRES-Cre mice (Left) and a representative coronal section showing the expression of the hM4D (Gi)-mCherry virus (Right). The white square is enlarged and displayed in **(C)**. Scale bar: 200 μm. **(C)** The triple-labeling immunofluorescence histochemical staining of c-FOS (green), hM4D (Gi)-mCherry (red) and nuclear dye DAPI (blue), showing that the expression of c-FOS in the Vpdm was lower in the CNO-injected group than in the saline-injected group (Saline: *n* = 3; CNO: *n* = 3). The double arrowheads indicate c-FOS/mCherry/DAPI triple-labeled neurons. The arrows indicate c-FOS/DAPI double-labeled neurons. And the single arrowheads indicate the mCherry/DAPI double-labeled neurons. Scale bar: 20 μm. **(D)** The total distance traveled in the OFT was identical under chemogenetic manipulation. **(E)** The inhibition of Vpdm^VGLUT1^ neurons increased the percentage of central distance and the time spent in the central area in the OFT. **(F)** The deactivation of Vpdm^VGLUT1^ neurons increased the percentage of open arm entries and the time spent in the open arms in the EPM. Data are expressed as the mean ± SEM. Tukey’s *post hoc* was performed between the groups: mCherry-CNO-treated control mice (*n* = 6) versus mCherry-CNO-treated UAC mice (*n* = 6), hM4Di-SAL-treated control mice (*n* = 8) versus hM4Di-SAL-treated UAC mice (*n* = 8), hM4Di-CNO-treated control mice (*n* = 7) versus hM4Di-CNO-treated UAC mice (*n* = 8), mCherry-CNO-treated UAC mice versus hM4Di-CNO-treated UAC mice and hM4Di-SAL-treated UAC mice versus hM4Di-CNO-treated UAC mice. *P < 0.05, **P < 0.01, ***P < 0.001.

**FIGURE 3:**
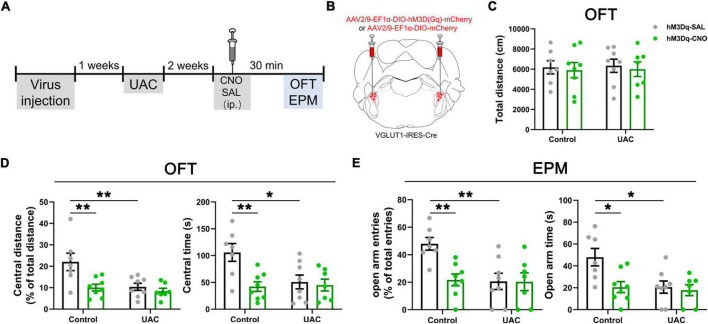
Chemogenetic activation of VGLUT1-ir neurons in the Vpdm induced anxiety-like behaviors in control mice but did not aggravate UAC-induced anxiety comorbidity. **(A)** Timeline of chemogenetic activation of Vpdm^VGLUT1^ neurons to investigate anxiety-like behaviors. **(B)** Schematic showing the virus injection into the Vpdm of VGLUT1-IRES-Cre mice. **(C)** The total distance traveled in the OFT was identical under chemogenetic manipulation. **(D)** In the OFT, the activation of Vpdm^VGLUT1^ neurons decreased the percentage of central distance and the time spent in the central area in the control mice but did not exacerbate UAC-induced anxiety comorbidity. **(E)** In the EPM, the activation of Vpdm^VGLUT1^ neurons decreased the percentage of open arm entries and the time spent in the open arms in the control mice but did not exacerbate UAC-induced anxiety comorbidity. Data are expressed as the mean ± SEM. Tukey’s *post hoc* was performed between the groups: hM3Dq-SAL-treated control mice (*n* = 7) versus hM3Dq-SAL-treated UAC mice (*n* = 8) and hM3Dq-SAL-treated control mice versus hM3Dq-CNO-treated control mice (*n* = 8). *P < 0.05, **P < 0.01.

**FIGURE 4:**
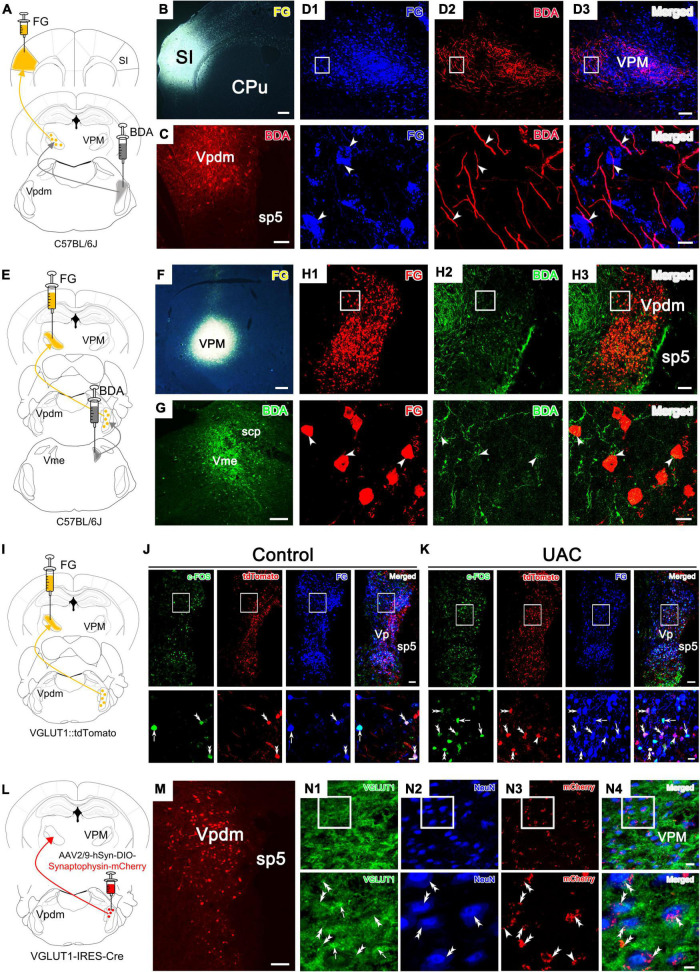
Tracing experiments with the Vpdm^VGLUT1^-VPM, Vpdm-VPM-SI and Vme-Vpdm-VPM pathways. **(A)** Schematic diagram of the Vpdm-VPM-SI neural pathway, demonstrating that FG was injected into the SI and BDA was injected into the Vpdm. **(B,C)** Representative coronal section showing the FG injection site in the SI and the BDA injection site in the Vpdm. Scale bar: 200 μm. **(D)** (Top) Immunofluorescence histochemical staining showing FG/BDA double-labeled neurons and terminals in the VPM. Scale bar: 100 μm. (Bottom) The enlarged VPM in the white square in D1-D3, showing that BDA-labeled nerve axons pass close to FG-retrograde-labeled neurons (arrowheads). Scale bar: 20 μm. **(E)** Schematic diagram of the Vme-Vpdm-VPM neural pathway, verifying that FG was injected into the VPM and BDA was injected into the Vme. **(F,G)** The FG injection site in the VPM and the BDA injection site in the Vme. Scale bar: 200 μm. **(H)** (Top) Immunofluorescence histochemical staining showing FG/BDA double-labeled neurons and terminals in the Vpdm. Scale bar: 100 μm. (Bottom) The enlarged Vpdm in the white square in H1–H3, showing that BDA-labeled axon terminals were close to FG-retrograde-labeled neuronal cell bodies (arrowheads). Scale bar: 20 μm. **(I)** Schematic diagram of the Vpdm-VPM pathway, demonstrating FG injection into the VPM of VGLUT1:tdTomato mice. **(J,K)** Top: The c-FOS/tdTomato/FG triple-labeled neurons in the Vpdm in the control mice **(J)** and the 2-week UAC mice **(K)**. Scale bar: 200 μm. Bottom: The enlarged triple-labeled neurons in the white square, with the double arrowheads indicating c-FOS/tdTomato/FG triple-labeled neurons. The arrows indicate c-FOS/FG double-labeled neurons. And the single arrowheads indicate the tdTomato/FG double-labeled neurons. Scale bar: 20 μm. **(L)** Schematic diagram verifying the Vpdm^VGLUT1^-VPM pathway by AAV2/9-hSyn-DIO-Synaptophysin-mCherry injection into the Vpdm of VGLUT1-IRES-Cre mice. **(M)** The expression of the synaptophysin-mCherry injection site in the Vpdm. Scale bar: 200 μm. **(N1–N4)** (Top) Triple-immunofluorescence histochemical staining of VGLUT1/NeuN/mCherry in the VPM showed that synaptophysin-mCherry-labeled terminals were coexpressed with VGLUT1 and were observed in close apposition on the NeuN-labeled VPM cell bodies. The double arrowheads indicate VGLUT1 + /mCherry-terminals near NeuN-labeled cell bodies, while single arrowheads indicate VGLUT1-/mCherry-terminals near NeuN-labeled cell bodies. The arrows indicate areas of intense VGLUT1 immunostaining that does not colocalize with mCherry-terminals. Scale bar: 20 μm. (Bottom) The enlarged area in the white square in **(N1–N4)**. VGLUT1/mCherry double-labeled terminals were in close contact with NeuN-labeled neuronal cell bodies (double arrowhead). Scale bar: 5 μm.

### Fluorescence *in situ* hybridization

Fluorescence *in situ* hybridization (FISH) was performed as previously described (Zhang C. K. et al., 2018). The VGLUT1:tdTomato mice were perfused and dissected as described above, and the brains were cut into 30 μm thick transverse sections. The target sections were used for FISH and immunofluorescence histochemistry. In brief, the sections were treated with 2% H_2_O_2_ in 0.1 M of PB for 10 min. After rinsing, the sections were incubated in 0.3% Triton-X100 for 20 min. The sections were then incubated for 10 min in acetylation solution containing 0.25% (v/v) acetic anhydride in 0.1 M triethanolamine. After rinsing, the sections were prehybridized for 1 h at 58°C. VGLUT1 riboprobes were subsequently added to the hybridization system at a final concentration of 1 μg/ml and incubated at 58°C for 20 h. After rinsing with wash buffer, the hybridized sections were incubated with 20 μg/ml ribonuclease for 30 min at 37°C and then rinsed in 0.2 × SSC. The sections were then incubated overnight at RT with 0.5 μg/ml peroxidase-conjugated anti-digoxigenin sheep antibody (11-207-733-910; Roche Diagnostics, Basel, Switzerland) and rabbit anti-RFP antibody (1:200; ab62341, Abcam). To amplify the VGLUT1 hybridization signals, we performed the biotinylated tyramine (BT)-glucose oxidase (GO) amplification method for 30 min. The sections were subsequently treated with 10 μg/mL Alexa Fluor 488-conjugated streptavidin (S11223, Invitrogen) and Alexa 594-conjugated donkey anti-rabbit IgG (1:500) in TSB for 3 h. Then, the sections were mounted on glass slides and observed under a confocal laser scanning microscope.

### Cell counting

In the hM4Di-induced Vpdm^VGLUT1^ inhibition experiment, six UAC mice were randomly divided into hM4Di-Saline-treated (*n* = 3) and hM4Di-CNO-treated groups (*n* = 3). The activity of the hM4Di-labeled neurons was verified by the ratio of the number of c-FOS/mCherry double-stained neurons to the number of mCherry-containing neurons (c-FOS + mCherry/mCherry). To verify the specificity of the VGLUT1:tdTomato mouse experiment, three mice were used in FISH evaluations. Six VGLUT1:tdTomato mice were randomly divided into 2-week control and UAC groups (*n* = 3/group). The activation of Vpdm^VGLUT1^ neurons and the Vpdm^VGLUT1^-VPM pathway was indicated by higher c-FOS + tdTomato/tdTomato ratios (the ratio of c-FOS/tdTomato double-labeled neurons to mCherry-labeled neurons) and c-FOS + tdTomato + FG/FG ratios (the ratio of c-FOS/tdTomato/FG triple-stained neurons to FG-labeled neurons) in 2-week UAC mice. In the activation of the Vpdm-VPM pathway via hM3Dq virus experiments, 6 control mice were randomly divided into hM3Dq-Saline-treated (*n* = 3) and hM3Dq-CNO-treated groups (*n* = 3). A higher ratio of c-FOS/mCherry double-stained neurons to mCherry-labeled neurons (c-FOS + mCherry/mCherry) was observed after CNO injection. In the above experiments, the brain slices (25 μm/slice) were collected sequentially in a six-well plate (about 5 slices in each well, 6 wells). For these counts, 5 brain slices covering the Vpdm from one well were selected and summarized as data for each mouse.

### Statistical analysis

All data are presented as the mean ± standard error of the mean (SEM). Statistical analyses were performed using GraphPad Prism 8 (Graph Pad Software, Inc., La Jolla, CA, USA). The data passed the normality and homogeneity of variance tests (Supplementary Tables 1–5). The c-FOS expression was analyzed *via* unpaired Student’s *t*-tests. The behavioral tests at different times and the chemogenetic data were analyzed by two-way ANOVA. The optogenetics data were analyzed by repeated measures two-way ANOVA (RM-ANOVA). Statistical significance was assessed by *post hoc* comparisons using Tukey tests. Statistical significance is indicated as *p < 0.05, ^**^p < 0.01, ^***^p < 0.001 and ^****^p < 0.0001.

## Results

### Mice with malocclusion showed anxiety-like behaviors

To examine the effect of UAC on negative emotions in mice, we first established a UAC model (Supplementary Figure 1A) and assessed anxiety-like behaviors at 1 week, 2 weeks, 4 weeks and 6 weeks with the OFT and EPM. The behavioral test results demonstrated that no significant anxiety-like behaviors were observed after 1 week of UAC (*p* > 0.05, Figures 1A–G). Two-way ANOVA revealed UAC effects [OFT: central distance, *F*_(1,51)_ = 36.31, *p* < 0.0001; central time, *F*_(1,51)_ = 37.41, *p* < 0.0001, Figures 1D, E and Table 1; EPM: open arm entries, *F*_(1,51)_ = 28.82, *p* < 0.0001; open arm time, *F*_(1,51)_ = 27.31, *p* < 0.0001, Figures 1F, G and Table 1]. Intriguingly, anxiety-like behaviors were significantly noticeable after 2 weeks of UAC and persisted for 6 weeks. Mice with UAC exhibited a lower percentage of central distance traveled than control mice (distance traveled in the central area versus the total distance; *p* = 0.0451, 2 weeks; *p* = 0.0114, 4 weeks; *p* = 0.0390, 6 weeks; Figure 1D) and spent significantly less time in the central zone after 2 weeks, 4 weeks and 6 weeks (*p* = 0.0078, 2 weeks; *p* = 0.0081, 4 weeks; *p* = 0.0258, 6 weeks; Figure 1E). Similarly, in the EPM, the percentage of open arm entries (open arm entries versus total entries; *p* = 0.0152, 2 weeks; *p* = 0.0398, 4 weeks; *p* = 0.0446, 6 weeks; Figure 1F) and the time spent in the open arms (*p* = 0.0362, 2 weeks; *p* = 0.0376, 4 weeks; *p* = 0.0256, 6 weeks; Figure 1G) were lower in the UAC group than in the control group. However, the total distance traveled in the OFT was not significantly different between control and UAC-treated mice (*p* > 0.05, Figure 1C and Table 1), demonstrating that UAC did not impair the locomotor activities of mice. According to the above behavioral results, the following anxiety-like behavioral assessments and c-FOS staining were conducted after 2 weeks of UAC.

**TABLE 1:** Data of behavioral tests in Figure 1 are given as mean ± standard error of mean.

	**OFT**						**EPM**			
	**Total distance**		**Central distance %**		**Central time**		**Open arm entries %**		**Open arm time**	
	**Con**	**UAC**	**Con**	**UAC**	**Con**	**UAC**	**Con**	**UAC**	**Con**	**UAC**
1 weeks	6072 ± 766.8	6204 ± 775.9	22.3 ± 2.56	16.8 ± 1.73	98.0 ± 9.70	82.2 ± 8.38	35.0 ± 3.58	30.4 ± 3.80	43.8 ± 6.36	41.5 ± 4.21
2 weeks	5565 ± 447.7	6487 ± 809.6	21.8 ± 2.13	12.1 ± 1.61	107.0 ± 12.55	50.0 ± 12.53	38.1 ± 4.55	14.0 ± 3.99	44.8 ± 5.91	16.2 ± 4.80
4 weeks	5840 ± 833.2	6252 ± 927.6	21.7 ± 2.91	10.8 ± 1.43	112.6 ± 7.38	57.6 ± 9.30	34.2 ± 6.69	13.6 ± 3.20	42.1 ± 7.98	14.4 ± 3.76
6 weeks	6125 ± 961.7	6195 ± 751.8	22.0 ± 2.01	12.4 ± 2.21	110.0 ± 11.1	60.8 ± 9.83	34.5 ± 5.87	13.7 ± 4.12	44.1 ± 8.02	15.2 ± 4.63

### Vpdm^VGLUT1^ neurons bidirectionally modulate anxiety comorbidity

Previous studies have reported elevated VGLUT1 expression in the Vpdm of animals with orofacial disorders and malocclusion-induced negative emotions (Liu et al., 2019; Shi et al., 2021). To determine whether VGLUT1-ir neurons in the Vpdm (Vpdm^VGLUT1^) participate in malocclusion-induced anxiety comorbidity, we employed designer receptors exclusively activated by designer drugs (DREADDs) to explore the effect of Vpdm^VGLUT1^ in modulating UAC-induced anxiety-like behaviors (Figure 2A). An adeno-associated virus (AAV) expressing the engineered Gi-coupled receptor [AAV2/9- Ef1α-DIO-hM4D (Gi)-mCherry] or a control virus (AAV2/9- Ef1α-DIO-mCherry) was stereotactically injected into the bilateral Vpdm of VGLUT1-IRES-Cre mice (Figure 2B and Supplementary Figures 1B–D). To verify that Vpdm^VGLUT1^ neurons were effectively inhibited after clozapine-N-oxide (CNO) administration, triple-staining for c-FOS/mCherry/DAPI was conducted. As shown in Figure 2C, fewer c-FOS/mCherry/DAPI triple-labeled neurons were observed in hM4Di-CNO mice than in hM4Di-Saline (hM4Di-SAL) mice (*p* = 0.0009, Figure 2C). After 2 weeks of UAC, 3 mg/kg CNO was intraperitoneally injected into the mice, and behavioral assessments were performed 30 min later. The total distance traveled in the OFT (Figure 2D and Table 2) did not differ significantly among the hM4Di-CNO, hM4Di-SAL and mCherry-CNO groups, demonstrating that the treatments did not impact the general locomotor abilities of the mice. Two-way ANOVA revealed UAC × hM4Di-CNO interaction effects [OFT: central distance, *F*_(2,37)_ = 7.224, *p* = 0.0022; central time, *F*_(2,37)_ = 5.228, *p* = 0.0100, Figure 2E and Table 2; EPM: open arm entries, *F*_(2,37)_ = 3.319, *p* = 0.0472; open arm time, *F*_(2,37)_ = 3.660, *p* = 0.0355, Figure 2F and Table 2]. In the UAC groups, *post hoc* analyses revealed that inhibition of VGLUT1-ir neurons in the Vpdm (hM4Di-CNO group) increased the percentage of central distance traveled (*vs.* mCherry-CNO, *p* = 0.0011; *vs.* hM4Di-SAL, *p* = 0.0012, Figure 2E) and the time spent in the central area (*vs.* mCherry-CNO, *p* = 0.0302; *vs.* hM4Di-SAL, *p* = 0.0493, Figure 2E) in the OFT. Similarly, in the EPM test, the percentage of open arm entries (*vs.* mCherry-CNO, *p* = 0.0120; *vs.* hM4Di-SAL, *p* = 0.0170, Figure 2F) and the time spent in the open arms (*vs.* mCherry-CNO, *p* = 0.0055; *vs.* hM4Di-SAL, *p* = 0.0071, Figure 2F) were significantly increased after Vpdm^VGLUT1^ inhibition. These results suggest that the inhibition of VGLUT1-ir neurons in the Vpdm produces anxiolytic effects. Moreover, anxiety-like behaviors were not alleviated in the hM4Di-SAL and mCherry-CNO groups, which indicates that chemogenetic operations and CNO injections have no effect on the anxiety-like behaviors induced by UAC.

**TABLE 2:** Data of behavioral tests in Figure 2 are given as mean ± standard error of mean.

	**OFT**						**EPM**			
	**Total distance**		**Central distance %**		**Central time**		**Open arm entries %**		**Open arm time**	
	**Con**	**UAC**	**Con**	**UAC**	**Con**	**UAC**	**Con**	**UAC**	**Con**	**UAC**
mCherry-CNO	5896 ± 722.5	5576 ± 842.4	24.8 ± 2.09	9.3 ± 1.71	110.9 ± 11.50	46.4 ± 11.68	42.9 ± 4.32	15.8 ± 5.36	43.2 ± 5.70	14.8 ± 3.68
hM4Di-SAL	5849 ± 702.8	6160 ± 780.1	20.0 ± 1.34	10.3 ± 1.72	113.8 ± 17.15	54.7 ± 11.79	42.1 ± 7.39	18.8 ± 4.66	40.5 ± 7.35	17.8 ± 4.06
hM4Di-CNO	6055 ± 1022	5808 ± 683.7	22.0 ± 1.82	22.5 ± 3.03	95.6 ± 17.18	109.6 ± 8.90	45.3 ± 5.27	43.3 ± 3.89	45.5 ± 5.04	45.6 ± 5.48

Furthermore, to test whether Vpdm^VGLUT1^ neurons are sufficient for anxiety comorbidity, we sought to selectively activate these neurons through DREADDs (Figure 3A). An AAV expressing the engineered Gq-coupled receptor [AAV2/9- Ef1α-DIO-hM3D (Gq)-mCherry] or a control virus was bilaterally injected into the Vpdm of VGLUT1-IRES-Cre mice (Figure 3B). After the hM3D (Gq) incubation and 2 weeks of UAC, the mice were injected with saline or CNO (3 mg/kg) and evaluated using the OFT and the EPM. The locomotor activities did not differ among the four groups (Figure 3C and Table 3). Two-way ANOVA revealed significant effects of hM3Dq-CNO on anxiety comorbidity [OFT: central distance, *F*_(1,26)_ = 4.594, *p* = 0.0416; central time, *F*_(1,26)_ = 5.324, *p* = 0.0293, Figure 3D and Table 3; EPM: open arm entries, *F*_(1,26)_ = 5.871, *p* = 0.0227; open arm time, *F*_(1,26)_ = 4.246, *p* = 0.0495, Figure 3E and Table 3]. In control mice, *post hoc* analyses revealed that hM3Dq-CNO treatment reduced the percentage of central distance (*vs.* hM3Dq-SAL, *p* = 0.0061, Figure 3D) and the time spent in the central area in the OFT (*vs.* hM3Dq-SAL, *p* = 0.0070, Figure 3D). In the EPM, this manipulation decreased the percentage of open arm entries (*vs.* hM3Dq-SAL, *p* = 0.0096, Figure 3E) and the time spent in the open arms (*vs.* hM3Dq-SAL, *p* = 0.0156, Figure 3E). However, in UAC mice, the activation of Vpdm^VGLUT1^ neurons did not aggravate the anxiety comorbidity (Figures 3D, E). Overall, the Vpdm^VGLUT1^ neuronal activity is necessary and sufficient to produce anxiety comorbidity.

**TABLE 3:** Data of behavioral tests in Figure 3 are given as mean ± standard error of mean.

	**OFT**						**EPM**			
	**Total distance**		**Central distance %**		**Central time**		**Open arm entries %**		**Open arm time**	
	**Con**	**UAC**	**Con**	**UAC**	**Con**	**UAC**	**Con**	**UAC**	**Con**	**UAC**
hM3Dq-SAL	6188 ± 648.2	6343 ± 652.5	22.1 ± 4.06	10.4 ± 1.64	105.8 ± 16.6	50.8 ± 12.94	48.0 ± 4.61	20.6 ± 5.88	48.0 ± 7.96	20.6 ± 5.57
hM3Dq-CNO	5903 ± 755.6	6005 ± 722.5	10.1 ± 1.54	8.4 ± 1.33	42.2 ± 8.89	45.1 ± 11.03	21.7 ± 4.29	20.4 ± 6.46	20.6 ± 5.08	17.8 ± 4.99

### The Vpdm^VGLUT1^ to VPM pathway participates in the modulation of UAC

We focused on an ascending pathway, namely, the trigeminal-thalamic-cortex pathway, which might relay orofacial information from trigeminal nuclei to the cortex. The VPM is the main target of the Vpdm (Smith, 1975; Ge et al., 2010; Andrew et al., 2020) and connects reciprocally with the SI (Oda et al., 2004; Kropf et al., 2019; Reuss et al., 2020). Previous reports have demonstrated that the Vpdm-VPM and VPM-SI pathways both participate in the modulation of proprioceptive disorders and negative emotion (Capra et al., 1994; Ge et al., 2010; Sato et al., 2017; Yoshida et al., 2017; Tsutsumi et al., 2018). To further confirm the existence of the Vpdm-VPM-SI pathway, we injected BDA into the Vpdm and FG into the SI (Figures 4A–C). Seven days later, double staining of BDA-labeled axons and terminals and FG-labeled neurons was observed in the VPM. Many BDA-labeled axons pass close to the FG-labeled cell bodies, indicating the possible existence of an anatomical pathway, namely, the Vpdm-VPM-SI pathway (Figures 4D1–D3). Consistent with previous studies (Zhang et al., 2001, 2005; Pang et al., 2006; Ge et al., 2010), we confirmed the putative links between the Vme and the Vpdm-VPM by injecting BDA into the Vme and FG into the VPM (Figures 4E–G and Supplementary Figures 2A, B). FG-labeled neurons and BDA-labeled axon terminals were observed in the Vpdm, and some BDA-labeled terminals were found in close apposition on the FG-labeled cell bodies (Figures 4H1–H3). We also injected FG into the Vpdm to further validate the Vme-Vpdm pathway. FG-labeled neurons were observed in the Vme but not in the parabrachial nucleus (Supplementary Figures 2C–E). These tracing results indicate the possible existence of ascending pathways: the Vme-Vpdm-VPM pathway and the Vpdm-VPM-SI pathway. Given the critical role of Vpdm^VGLUT1^ neurons in the modulation of UAC-induced anxiety-like behaviors, we investigated whether the Vpdm^VGLUT1^-VPM pathway participated in 2-week UAC. To observe VGLUT1-ir neurons in the Vpdm, we crossed a VGLUT1-IRES-Cre mouse line with the Cre-dependent tdTomato mouse line Ai9 (VGLUT1:tdTomato mice). We first validated that VGLUT1:tdTomato mice selectively express red fluorescent protein (RFP) in response to Vpdm^VGLUT1^ neurons by FISH for VGLUT1 mRNA and immunostaining for RFP. In VGLUT1:tdTomato mice, 90.0% of VGLUT1 mRNA-positive cells were RFP positive (Supplementary Figure 2G; 360 RFP and VGLUT1 mRNA double-positive cells out of 400 VGLUT1 mRNA-positive cells), and 92.5% of RFP-positive cells were VGLUT1 mRNA-positive in the Vpdm (Supplementary Figure 2H; 360 RFP and VGLUT1 mRNA double-positive cells out of 389 RFP-positive cells). Then, FG was injected into the VPM of VGLUT1:tdTomato mice (Figure 4I). To determine whether the Vpdm^VGLUT1^ and Vpdm^VGLUT1^-VPM pathways participated in the 2-week UAC, triple-immunofluorescence histochemical staining of c-FOS/FG/tdTomato was performed in the Vpdm. The activities of Vpdm^VGLUT1^ neurons were assessed according to the ratio of c-FOS/tdTomato double-stained neurons to tdTomato-labeled neurons (c-FOS + tdT/tdT) (Figures 4J, K). The c-FOS + tdT/tdT ratio in the UAC group was higher than that in the control group (*p* = 0.0002, Supplementary Figure 2I). Similarly, the activation of the VGLUT1-ir neurons contained in the Vpdm^VGLUT1^-VPM pathway in UAC mice was determined according to the higher ratio of c-FOS + tdT + FG/tdT + FG (the ratio of c-FOS/tdTomato/FG triple-stained neurons to tdTomato/FG double-labeled neurons) than that in the control group (*p* = 0.0002, Supplementary Figure 2J). To further determine the activity of VGLUT1-ir neurons and non-VGLUT1-ir neurons contained in the Vpdm-VPM pathway, the higher ratio of c-FOS + tdT + FG/FG (*p* = 0.0007, Supplementary Figure 2K) and c-FOS + FG (tdT-)/FG (*p* = 0.0008, Supplementary Figure 2L) were found in the UAC mice, which indicated that the activity of VGLUT1-ir and non-VGLUT1-ir neurons was increased in the 2-week UAC mice. To identify the synaptic connectivity between Vpdm^VGLUT1^ neurons and the VPM, an anterograde AAV expressing Cre-dependent synaptophysin-mCherry (AAV2/9-hSyn-DIO-Synaptophysin-mCherry) was injected into the Vpdm of the VGLUT1-IRES-Cre mice (Figure 4L). After 3 weeks of virus incubation, the viral injection site in the Vpdm was observed, as shown in Figure 4M, and synaptophysin-mCherry-labeled terminals were detected in the VPM (Figure 4N3). To determine whether the synaptophysin-mCherry-labeled terminals were VGLUT1, triple-staining for VGLUT1/NeuN/mCherry was conducted in the VPM (Figures 4N1–N4). Abundant synaptophysin-mCherry-labeled terminals were coexpressed with VGLUT1 and observed close to NeuN-labeled VPM cell bodies, indicating Vpdm^VGLUT1^-targeted VPM neurons. However, there also existed mCherry-labeled terminals that do not label with VGLUT1-immunofluorescence. These neurons containing only mCherry-labeled terminals are presumed as co-expression of VGLUT1 and VGLUT2 mRNA neurons, which terminals lacking VGLUT1 staining are expressing primarily VGLUT2. These tracing studies suggest the existence of putatively ascending pathways, namely, the Vme-Vpdm-VPM and Vpdm-VPM-SI pathways. Moreover, 2-week UAC led to hyperactivity in Vpdm^VGLUT1^ neurons and the Vpdm^VGLUT1^-VPM pathway.

### Vpdm-VPM-projecting neurons bidirectionally modulate anxiety-like behaviors in UAC mice

**FIGURE 5:**
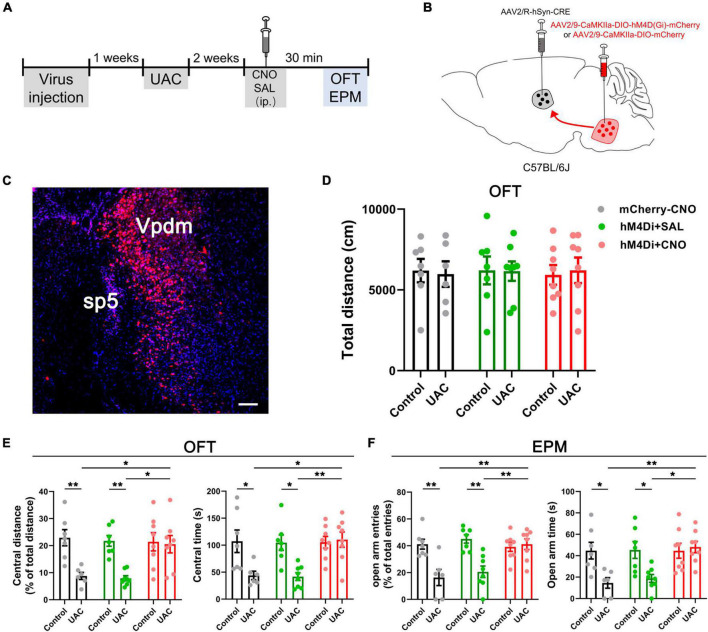
Chemogenetic inhibition of the Vpdm-VPM pathway alleviated anxiety-related behaviors induced by UAC. **(A)** Experimental design of the chemogenetic inhibition of the Vpdm-VPM pathway to investigate anxiety-related behaviors. **(B)** Virus injection strategy. **(C)** Representative coronal section showing the expression of the hM4D (Gi)-mCherry virus in the Vpdm. Scale bar: 200 μm. **(D)** The total distance traveled in the OFT was identical among the different groups. **(E)** The inhibition of the Vpdm-VPM pathway increased the percentage of central distance and time spent in central area in the OFT. **(F)** The deactivation of the Vpdm-VPM pathway increased the percentage of open arm entries and the time spent in the open arms in the EPM. Data are expressed as the mean ± SEM. Tukey’s *post hoc* was performed between the groups: mCherry-CNO-treated control mice (*n* = 7) versus mCherry-CNO-treated UAC mice (*n* = 6), hM4Di-SAL-treated control mice (*n* = 7) versus hM4Di-SAL-treated UAC mice (*n* = 8), hM4Di-CNO-treated control mice (*n* = 8) versus hM4Di-CNO-treated UAC mice (*n* = 8), mCherry-CNO-treated UAC mice versus hM4Di-CNO-treated UAC mice and hM4Di-SAL-treated UAC mice (*n* = 8) versus hM4Di-CNO-treated UAC mice. *P < 0.05, **P < 0.01.

We next investigated whether the Vpdm-VPM pathway played a necessary role in comorbid anxiety. In wild-type mice, an inhibitory chemogenetic virus [AAV2/9-CaMKIIa-DIO-hM4D(Gi)-mCherry] or a control virus (AAV2/9-CaMKIIa-DIO-mCherry) was injected into the bilateral Vpdm, and a retrogradely transported AAV expressing a Cre recombinase (AAV2/R-hSyn-Cre) was bilaterally injected into the VPM (Figures 5A–C). As shown in Figure 4A, after 2 weeks of UAC, behavioral assessments were conducted. Chemogenetic inhibition of Vpdm-VPM-projecting neurons did not have a substantial effect on the total distance traveled in the OFT (Figure 5D and Table 4). However, two-way ANOVA demonstrated considerable UAC × hM4Di-CNO interaction effects [OFT: central distance, *F*_(2,38)_ = 4.461, *p* = 0.0182; central time, *F*_(2,38)_ = 4.535, *p* = 0.0171, Figure 5E and Table 4; EPM: open arm entries, *F*_(2,38)_ = 7.416, *p* = 0.0019; open arm time, *F*_(2,38)_ = 4.450, *p* = 0.0183, Figure 5F and Table 4]. The inhibition of Vpdm-VPM-projecting neurons (hM4Di-CNO group) increased percentage of central distance (*vs.* mCherry-CNO, *p* = 0.0380; *vs.* hM4Di-SAL, *p* = 0.0105, Figure 5E) and the time spent in the central zone (*vs.* mCherry-CNO, *p* = 0.0178; *vs.* hM4Di-SAL, *p* = 0.0061, Figure 5E) in the OFT. In the EPM, the inhibition of Vpdm-VPM-projecting neurons increased the percentage of the open arm entries (*vs.* mCherry-CNO, *p* = 0.0022; *vs.* hM4Di-SAL, *p* = 0.0075, Figure 5F) and the time spent in the open arms (*vs.* mCherry-CNO, *p* = 0.0094; *vs.* hM4Di-SAL, *p* = 0.0163, Figure 5F). Moreover, the chemogenetic virus (hM4Di-SAL group) and CNO injection (mCherry-CNO group) did not have substantial effects on locomotor abilities or anxiety-like behaviors. These chemogenetic results reveal that specific inhibition of Vpdm-VPM-projecting neurons produces antianxiety effects in UAC mice.

**TABLE 4:** Data of behavioral tests in Figure 5 are given as mean ± standard error of mean.

	**OFT**						**EPM**			
	**Total distance**		**Central distance %**		**Central time**		**Open arm entries %**		**Open arm time**	
	**Con**	**UAC**	**Con**	**UAC**	**Con**	**UAC**	**Con**	**UAC**	**Con**	**UAC**
mCherry-CNO	6197 ± 723.9	5981 ± 791.9	22.9 ± 2.98	8.8 ± 1.31	107.3 ± 20.78	43.9 ± 7.77	41.2 ± 3.65	16.3 ± 6.01	44.7 ± 7.78	14.6 ± 4.90
hM4Di-SAL	6211 ± 864.0	6163 ± 596.9	21.8 ± 1.98	7.9 ± 0.95	104.6 ± 14.24	41.7 ± 7.15	45.1 ± 3.16	20.6 ± 4.31	45.3 ± 7.91	18.8 ± 3.69
hM4Di-CNO	5931 ± 608.3	6213 ± 789.2	21.4 ± 3.37	20.5 ± 3.22	105.4 ± 10.82	110.3 ± 14.21	39.2 ± 3.36	41.2 ± 3.99	44.7 ± 7.00	48.1 ± 5.31

**FIGURE 6:**
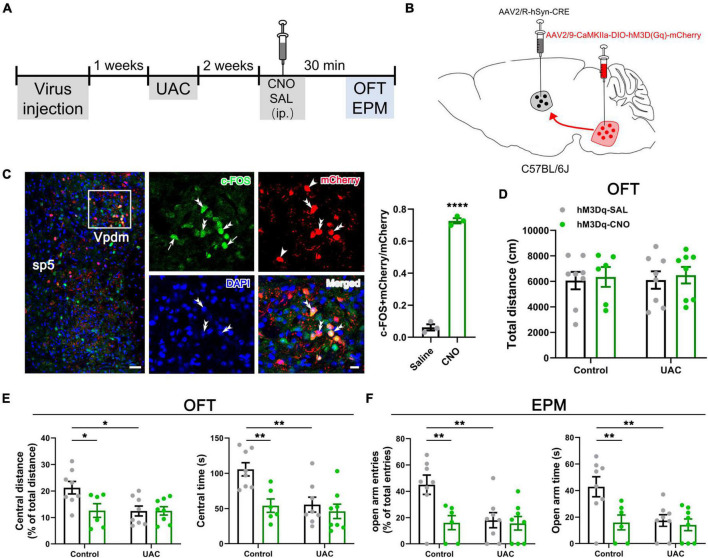
Chemogenetic2 activation of the Vpdm-VPM pathway induced anxiety-like behaviors in control mice but did not aggravate UAC-induced anxiety comorbidity. **(A)** Timeline of the chemogenetic activation of the Vpdm-VPM pathway in the behavioral tests. **(B)** Virus injection strategy. **(C)** (Left) Infection images of the hM3D (Gq)-mCherry virus. The white square is enlarged and displayed in the middle. Scale bar: 200 μm. (Middle) The c-FOS/hM3D(Gq)-mCherry/DAPI triple-labeled neurons after 150 min of CNO injections. The double arrowheads indicate triple-labeled neurons. The arrows indicate c-FOS/DAPI double-labeled neurons. And the single arrowheads indicate the mCherry/DAPI double-labeled neurons. Scale bar: 20 μm. (Right) The expression of c-FOS in the Vpdm was higher in the CNO-injected group than in the saline-injected group (Saline: *n* = 3; CNO: *n* = 3). **(D)** The general locomotor activities did not differ under various experimental manipulation conditions. **(E)** The activation of the Vpdm-VPM pathway decreased the percentage of central distance traveled and the time spent in the central area in the OFT in the control mice but did not exacerbate UAC-induced anxiety-like behaviors. **(F)** The activation of the Vpdm-VPM pathway decreased the percentage of open arm entries and the time spent in the open arms in the EPM in the control mice but did aggravate behaviors induced by UAC. Data are expressed as the mean ± SEM. Tukey’s *post hoc* was performed between the groups: hM3Dq-SAL-treated control mice (*n* = 8) versus hM3Dq-SAL-treated UAC mice (*n* = 8) and hM3Dq-SAL-treated control mice versus hM3Dq-CNO-treated control mice (*n* = 6). *P < 0.05, **P < 0.01, ****P < 0.0001.

Next, to test whether Vpdm-VPM-projecting neurons are sufficient for UAC-induced anxiety comorbidity, we sought to selectively activate these neurons through DREADDs (Figure 6A). AAV2/9-CaMKIIa-DIO-hM3D(Gq)-mCherry was microinjected into the bilateral Vpdm, and AAV2/R-hSyn-Cre was bilaterally delivered into the VPM (Figure 6B). A higher ratio of c-FOS/mCherry double-stained neurons to mCherry-labeled neurons (c-FOS + mCherry/mCherry) was observed in the Vpdm in the hM3Dq-CNO group than in the hM3Dq-SAL group (*p* < 0.0001, Figure 6C), which indicated the effective activation of Vpdm-VPM-projecting neurons. As shown in Figure 5A, after 3-week incubation of the virus, behavioral experiments were conducted. The locomotor activities did not differ among the different groups (Figure 6D and Table 5). Two-way ANOVA revealed significant effects of hM3Dq-CNO on anxiety-like behaviors [OFT: central distance, *F*_(1,26)_ = 4.250, *p* = 0.0494; central time, *F*_(1,26)_ = 4.320, *p* = 0.0477, Figure 6E and Table 5; EPM: open arm entries, *F*_(1,26)_ = 4.649, *p* = 0.0405; open arm time, *F*_(1,26)_ = 4.251, *p* = 0.0494, Figure 6F and Table 5]. In control mice, *post hoc* analyses revealed that hM3Dq-CNO treatment reduced the percentage of central distance (*vs.* hM3Dq-SAL, *p* = 0.0451, Figure 6E) and the time spent in the central region in the OFT (*vs.* hM3Dq-SAL, *p* = 0.0093, Figure 6E). Furthermore, in the EPM, this manipulation decreased the percentage of open arm entries (*vs.* hM3Dq-SAL, *p* = 0.0179, Figure 6F) and the time spent in the open arms (*vs.* hM3Dq-SAL, *p* = 0.0180, Figure 6F). However, in UAC mice, the activation of Vpdm-VPM-projecting neurons did not aggravate anxiety-like behaviors (Figures 6E, F). Overall, these results suggest that Vpdm-VPM-projecting neurons are crucial in UAC-induced anxiety-like behaviors.

**TABLE 5:** Data of behavioral tests in Figure 6 are given as mean ± standard error of mean.

	**OFT**						**EPM**			
	**Total distance**		**Central distance %**		**Central time**		**Open arm entries %**		**Open arm time**	
	**Con**	**UAC**	**Con**	**UAC**	**Con**	**UAC**	**Con**	**UAC**	**Con**	**UAC**
hM3Dq-SAL	6062 ± 681.1	6103 ± 683.1	21.3 ± 2.35	12.5 ± 1.87	105.7 ± 9.39	55.6 ± 10.52	45.1 ± 7.33	18.0 ± 5.78	42.9 ± 7.55	17.4 ± 4.41
hM3Dq-CNO	6384 ± 780.8	6491 ± 650.7	12.7 ± 2.58	12.4 ± 1.59	54.1 ± 9.51	46.1 ± 10.21	16.2 ± 5.40	15.7 ± 5.22	16.0 ± 5.68	14.2 ± 4.44

### Optogenetic inhibition of the Vpdm^VGLUT1^-VPM pathway reverses anxiety-like behaviors induced by UAC

**FIGURE 7:**
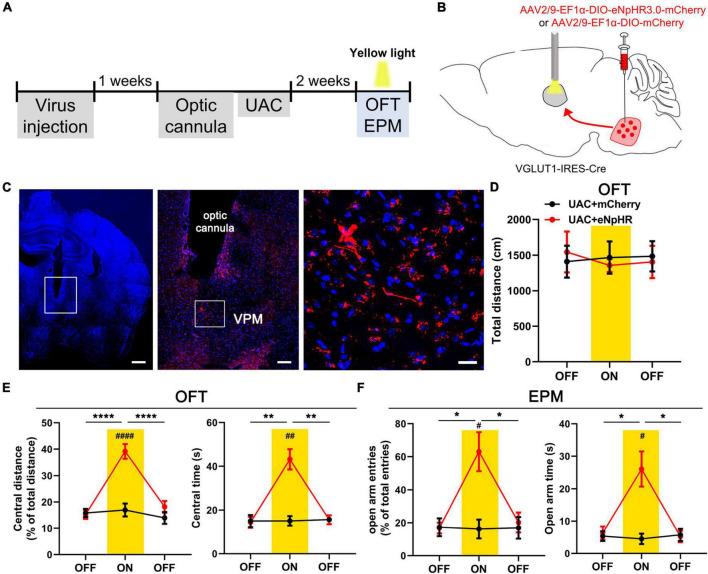
Optogenetic inhibition of the Vpdm^VGLUT1^-VPM pathway reverses anxiety comorbidity in UAC mice. **(A)** Timeline of the optogenetic inhibition of the Vpdm^VGLUT1^-VPM pathway in the behavioral assays. **(B)** Schematic showing the virus injection and the optic cannula implanted in the Vpdm of VGLUT1-IRES-Cre mice. **(C)** (Left) Representative coronal brain slice image of the VPM and implantation site of the optical fiber. Scale bar: 50 μm. (Middle) The enlarged area in the white square on the left side shows the expression of the eNpHR-mCherry terminal and the implantation site of the optical fiber in the VPM. Scale bar: 200 μm. (Right) The enlarged area in the white square in the middle shows the eNpHR-mCherry terminals in the VPM. Scale bar: 20 μm. **(D)** The total distance traveled in the OFT was identical with and without yellow laser stimulation. **(E)** Optogenetic inhibition of the Vpdm^VGLUT1^-VPM pathway increased the percentage of central distance traveled and the time spent in the central area in the OFT. **(F)** The deactivation of the Vpdm^VGLUT1^-VPM pathway increased the percentage of open arm entries and the time spent in the open arms in the EPM. Data are expressed as the mean ± SEM. Tukey’s *post hoc* was performed between the groups: eNpHR-ON UAC mice (*n* = 7) versus eNpHR-OFF UAC mice and eNpHR-ON UAC mice versus mCherry-ON UAC mice (*n* = 7). *P < 0.05, **P < 0.01, ****P < 0.0001.

To examine the role of VGLUT1-ir neurons in the Vpdm-VPM pathway on comorbid anxiety, we utilized optogenetics to inhibit the Vpdm^VGLUT1^-VPM pathway after 2 weeks of UAC. An AAV carrying inhibitory opsin halorhodopsin (AAV2/9- EF1α-DIO-eNpHR3.0-mCherry) or a control virus (AAV2/9-EF1a-DIO-mCherry) was bilaterally injected into the Vpdm of VGLUT1-IRES-Cre mice. Two weeks before behavioral assessments, optical fibers were implanted 200 μm above the VPM and the UAC model was established (Figures 7A–C). We observed that optogenetic silencing of the Vpdm^VGLUT1^-VPM pathway did not influence the general locomotor abilities of the mice (Figure 7D and Table 6). Moreover, in UAC mice, RM-ANOVA showed obvious effects of eNpHR light on anxiety-like behaviors [OFT: central distance, *F*_(2,24)_ = 14.63, *p* = 0 < 0001; central time, *F*_(2,24)_ = 20.74, *p* < 0.0001, Figure 7E and Table 6; EPM: open arm entries, *F*_(2,24)_ = 6.756, *p* = 0.0047; open arm time, *F*_(2,24)_ = 11.06, *p* = 0.0004, Figure 7F and Table 6]. *Post hoc* analyses showed that eNpHR light increased the percentage of central distance (*vs.* mCherry light, *p* < 0.0001, Figure 7E) and the time spent in the central area (*vs.* mCherry light, *p* = 0.0014, Figure 7E) in the OFT and elevated the percentage of open arm entries (*vs.* mCherry light, *p* = 0.0183, Figure 7F) and the time spent in open arms (*vs.* mCherry light, *p* = 0.0189, Figure 7F) in the EPM. In the control group, the optogenetic inhibition of the Vpdm^VGLUT1^-VPM pathway did not have significant effects on the locomotor activities or anxious states of the mice (Supplementary Figures 3A, B and Table 6). Overall, we demonstrated that silencing the Vpdm^VGLUT1^-VPM pathway mitigates UAC-induced anxiety-like behaviors but does not alter anxious states in control mice.

**TABLE 6:** Data of behavioral tests in Figure 7 and Supplementary Figure 3 are given as mean ± standard error of mean.

	**OFT**						**EPM**			
	**Total distance**		**Central distance %**		**Central time**		**Open arm entries %**		**Open arm time**	
	**Con**	**UAC**	**Con**	**UAC**	**Con**	**UAC**	**Con**	**UAC**	**Con**	**UAC**
eNpHR-OFF1	1428 ± 227.2	1545 ± 285.9	30.0 ± 3.68	15.3 ± 1.69	30.9 ± 4.47	14.4 ± 2.54	43.3 ± 11.99	16.8 ± 3.29	16.7 ± 4.65	6.3 ± 2.00
eNpHR-ON	1428 ± 159.4	1357 ± 91.1	32.5 ± 3.23	39.1 ± 2.79	34.4 ± 4.12	43.2 ± 4.64	41.9 ± 7.76	63.1 ± 11.73	16.3 ± 3.49	26.1 ± 5.40
eNpHR-OFF2	1595 ± 206.0	1405 ± 226.5	34.9 ± 3.23	18.2 ± 2.23	32.8 ± 5.44	15.6 ± 2.03	42.6 ± 8.36	20.2 ± 6.07	14.9 ± 3.19	5.2 ± 1.77
mCherry-OFF1	1540 ± 306.4	1410 ± 223.9	31.8 ± 6.47	15.9 ± 1.56	32.1 ± 4.23	15.0 ± 2.75	41.1 ± 13.66	17.3 ± 5.44	14.1 ± 5.11	5.3 ± 1.50
mCherry-ON	1491 ± 248.9	1467 ± 226.9	32.9 ± 4.08	17.0 ± 2.46	32.0 ± 5.26	15.1 ± 2.17	45.1 ± 9.89	16.2 ± 5.73	15.0 ± 4.74	4.5 ± 1.61
mCherry-OFF2	1506 ± 227.1	1485 ± 212.9	34.2 ± 3.77	14.0 ± 2.25	32.7 ± 6.34	15.7 ± 0.74	42.0 ± 9.01	16.9 ± 6.56	16.7 ± 4.38	5.7 ± 1.89

## Discussion

Many studies have demonstrated a strong relationship between malocclusion and anxiety disorders (Tang et al., 2017; Liu et al., 2019), which is a common symptom in TMD patients (Theroux et al., 2019). The potential central mechanism underlying this phenomenon has attracted widespread attention in neurological and dental sciences. In the present study, we observed that UAC induced anxiety-like behaviors. The inhibition of Vpdm^VGLUT1^ neurons reduced anxiety-like behaviors, while the activation of these neurons induced anxiety-like behaviors in control mice. Neural tract tracing demonstrated the putative existence of an ascending Vme-Vpdm^VGLUT1^-VPM-SI neural pathway, representing an essential neural substrate for anxiety-like behaviors induced by malocclusion. Furthermore, the inhibition of the Vpdm-VPM pathway in the UAC model ameliorated anxiety-like behaviors, while the activation of the neural pathway induced related behaviors in control mice.

In this study, we employed the UAC model to explore the influence of malocclusion on negative emotions. In addition to osteoarthritic lesions in the temporomandibular joints, UAC induces muscle injury and atrophic adaptive activity in the masseter muscle (Yang et al., 2020). Moreover, early removal of UAC results in general improvement or partial recovery of these changes (Jin et al., 2020). These works have revealed that occlusal disharmony impairs trigeminal motor function by activating the Vme. Consistent with previous studies, our results revealed that anxiety-like behaviors can be induced by 2-week, 4-week and 6-week UAC, which manifests as reduced time spent and distances traveled in the center zone in the OFT and decreased time and entries into open arms in the EPM (Tang et al., 2017; Liu et al., 2019).

The proprioceptive and affective information derived from orofacial disorders are transmitted via the trigemino-thalamic pathway (Diamond et al., 1992; Ge et al., 2014; Yoshida et al., 2017), which mainly arises from the Vpdm to the VPM (Veinante and Deschênes, 1999; Ge et al., 2010; Yoshida et al., 2017). About 64% glutamatergic neurons in the Vp coexpress VGLUT1 and VGLUT2 mRNA and other neurons express only VGLUT1 mRNA or VGLUT2 mRNA, respectively (Ge et al., 2010; Zhang C. K. et al., 2018). Some Vpdm-VPM-projecting neurons are double-labeled with VGLUT1 mRNA and VGLUT2 mRNA (Ge et al., 2010). Our c-FOS and tracing results demonstrated that 2-week UAC mice had higher ratios of triple labeled (c-FOS + /tdT + /FG +) to FG labeled neurons (Supplementary Figure 2K) and the double labeled (c-FOS + /tdT-/FG +) to FG labeled neurons (Supplementary Figure 2L) compared to control mice. This indicated that both VGLUT1-ir and non-VGLUT1-ir (putative VGLUT2-ir) neurons contained in the Vpdm-VPM pathway were activated by 2-weeks of UAC. The activation of Vpdm^VGLUT1^ neurons is critical for UAC induced anxiety comorbidity. According to previous studies, the activation of putative VGLUT2-ir neurons was related to the negative emotion (Taylor et al., 2019). Whether the Vpdm^VGLUT2^-VPM pathway modulated the anxiety comorbidity induced by UAC should be further studied.

Moreover, in the trigeminal pathway, the Vme and Vpdm have both been shown to transmit proprioception and the Vme express only VGLUT1-mRNA (Pang et al., 2006; Liu et al., 2018; Shi et al., 2021). In our previous studies, the expression of VGLUT1 in the Vme and Vpdm was upregulated under UAC conditions (Liu et al., 2017, 2018; Shi et al., 2021). Based on the above studies, we proposed that the hyperactivity of VGLUT1-ir neurons in the Vme-Vpdm pathway might play a pivotal role in the anxiety comorbidity induced by dental malocclusion. In this study, the deactivation of Vpdm^VGLUT1^ neurons by DREADDs returned UAC-induced anxiety-like behaviors to normal levels in the OFT and EPM. Further, activation of these neurons induced the anxiety-like behaviors in control mice but did not aggravate the anxiety comorbidity in UAC mice. These results suggest that VGLUT1-ir neurons in the Vpdm have a critical role in the modulation of anxiety comorbidity.

The VPM is the main target of the Vpdm (Smith, 1975; Ge et al., 2010; Andrew et al., 2020) and is also involved in the delivery of orofacial proprioceptive signals through trigemino-thalamic fibers (Capra et al., 1994; Yoshida et al., 2017). Some neurons in the Vme project to the Vpdm, and through these projections, proprioceptive information is transmitted ascendingly to the VPM (Zhang et al., 2001, 2005; Pang et al., 2006; Ge et al., 2010). A previous study reported that VGLUT1-ir and VGLUT2-ir neurons in the Vpdm both innervated the VPM and formed asymmetric synapses with dendritic structures of VPM neurons (Ge et al., 2010). In this study, by combining tracing results and c-FOS immunofluorescence staining in VGLUT1:tdTomato mice, we demonstrated the putative Vme-Vpdm-VPM pathway and the potential Vpdm^VGLUT1^-VPM pathway underlying comorbid anxiety, implying neural connections between proprioception and emotions. However, due to the limitations of fluorescence imaging we cannot provide definitive evidence for functional connections in the Vme-Vpdm-VPM and Vpdm-VPM-SI pathways. Electrophysiological recordings or electron-microscopy experiments should be conducted in the future.

Then, we focused on the involvement of the Vpdm^VGLUT1^-VPM pathway, a key ascending neural pathway, in UAC-induced anxiety disorders. Hyperactivation of the Vpdm^VGLUT1^-VPM pathway was verified to participate in 2-week UAC by the elevated ratio of c-FOS + tdT + FG/tdT + FG in the Vpdm of VGLUT1:tdTomato mice. Furthermore, inhibition of the Vpdm-VPM neural pathway by hM4D(Gi) mitigated anxiety comorbidity. However, under normal conditions, the activation of the Vpdm-VPM pathway via hM3D(Gq) induced anxiety-like behaviors. Consistently, antianxiety effects were observed after inhibiting the Vpdm^VGLUT1^-VPM pathway using optogenetics in VGLUT1-IRES-Cre mice. The above results demonstrate that the Vpdm^VGLUT1^-VPM pathway participates in the process of UAC-induced anxiety-like behaviors. The present results, together with previous reports, suggested that the Vme^VGLUT1^-Vpdm^VGLUT1^-VPM pathway might be involved in the generation and maintenance of malocclusion-induced anxiety comorbidity.

Dysfunction of the thalamocortical pathway is associated with proprioceptive disorders (Sato et al., 2017; Tsutsumi et al., 2018) and negative emotions (Ramsay, 2019; Sheffield et al., 2020; Wei et al., 2020). The SI, a well-known brain region that participates in emotional regulation (Kropf et al., 2019; Jin et al., 2020), is connected reciprocally with the VPM (Oda et al., 2004; Kropf et al., 2019; Reuss et al., 2020). In inflammatory pain-induced comorbid anxiety, enhanced excitation of the SI-dorsolateral striatum pathway has been observed (Jin et al., 2020). According to the present tracing result, the VPM receives afferent fibers derived from the Vpdm and projects to the SI. Thus, we propose that the potential Vpdm^VGLUT1^-VPM-SI pathway (Rausell and Jones, 1991) is involved in the modulation of malocclusion-induced anxiety symptoms. Moreover, widespread connections between the SI and the limbic system have been observed (Price, 2000), implying the potential influence of malocclusion on nuclei that mediate mood disorders. Further in-depth studies should explore the role of the SI in malocclusion-induced anxiety emotions.

Overall, the present study improves our understanding of the Vpdm^VGLUT1^-VPM pathway, which plays a critical role in malocclusion-induced anxiety comorbidity. Moreover, a potential trigeminal-thalamic-cortex pathway (Vme^VGLUT1^-Vpdm^VGLUT1^-VPM-SI) that may be involved in anxiety comorbidity was detected for the first time in our study. These findings provide novel insight into the relationship between malocclusion and negative emotions. Deactivation of this neural pathway could be an effective therapeutic strategy for mitigating malocclusion-induced negative emotions.

## Data availability statement

The original contributions presented in this study are included in the article/Supplementary material, further inquiries can be directed to the corresponding authors.

## Ethics statement

This animal study was reviewed and approved by Animal Care and Use Committees of the Fourth Military Medical University.

## Author contributions

Y-YJ, XLiu, M-QW, J-LL, and J-HL conceived and designed the experiments and wrote the manuscript. Y-YJ, XLiu, XLi, Y-FX, JW, and YF performed the experiments and acquired the data. Y-YJ, XLiu, TM, and JS analyzed the data. All authors discussed the manuscript, and read and approved the final manuscript.

## Supplementary material

Supplementary Figure 1 **(A)** Schematic diagram and photograph of the UAC model. A section of metal tube with a length of 1.5 mm and an inner diameter of 0.3 mm was bonded onto the rat left maxillary incisor. A curved section of metal tube with a length of 2.5 mm and an inner diameter of 0.3 mm was adhered to the left mandibular incisor. The mandibular tube was bent and tilted to form a 135° angle with the occlusal plate. **(B–D)** Representative coronal section showing the rostrocaudal expression of the virus in the Vpdm (coordinates relative to Bregma: –5.02 mm, –5.34 mm and –5.68 mm). Scale bar: 200 μm.

Supplementary Figure 2 **(A,B)** Representative coronal section showing the rostrocaudal expression of FG in the VPM (coordinates relative to Bregma –1.46 mm and –1.94 mm). Scale bar: 100 μm. **(C,D)** The Vme-Vpdm pathway was verified by injecting FG into the Vpdm. Scale bar: 200 μm. **(E)** FG-retrograde-labeled neuronal cell bodies were observed in the Vme. Scale bar: 100 μm. The enlarged FG-labeled neurons in the white square. Scale bar: 50 μm. **(F)** (Top) VGLUT1/RFP double-labeled neurons in the Vpdm in VGLUT1:tdTomato mice. Scale bar: 200 μm. (Bottom) The enlarged double-labeled neurons in the white square, with the double arrowheads indicating VGLUT1/RFP double-labeled neurons. The arrows indicate VGLUT1-labeled neurons. And the single arrowheads indicate the RFP -labeled neurons. Scale bar: 20 μm. **(G,H)** The pie chart shows the number of VGLUT1/RFP double-labeled neurons versus the total number of VGLUT1-labeled neurons and RFP-labeled neurons. **(I,J)** In VGLUT1:tdTomato mice, c-FOS/FG/tdTomato triple-stained neurons were observed in the 2-week control (*n* = 3) and UAC (*n* = 3) groups. **(I)** The activation of Vpdm^VGLUT1^ neurons in UAC mice was displayed by the higher c-FOS + tdT/tdT ratio (the ratio of c-FOS/tdTomato double-stained neurons to tdTomato-labeled neurons). **(J)** The activation of the VGLUT1-ir neurons contained in the Vpdm^VGLUT1^-VPM pathway in UAC mice was determined according to the higher c-FOS + tdT + FG/tdT + FG ratio (the ratio of c-FOS/tdTomato/FG triple-stained neurons to tdTomato/FG double-labeled neurons). **(K)** The activation of VGLUT1-ir neurons contained in the Vpdm-VPM pathway was displayed by the higher ratio of c-FOS + tdT + FG/FG in the UAC mice. **(L)** The activation of non-VGLUT1-ir neurons contained in the Vpdm-VPM pathway was determined according to the higher ratio of c-FOS + FG (tdT-)/FG in the UAC mice. Data are expressed as the mean ± SEM. ^***^*P* < 0.001.

Supplementary Figure 3 Optogenetic inhibition of the Vpdm^VGLUT1^-VPM pathway did not alter anxiety-like behaviors in the control group. **(A,B)** Optogenetic inhibition of the Vpdm^VGLUT1^-VPM pathway did not affect locomotor abilities or anxiety-like behaviors in the OFT and EPM. Data are expressed as the mean ± SEM. Control + mCherry: *n* = 6; Control + eNpHR: *n* = 7.
