# Supplementary figures and images for: Activation of the VpdmVGLUT1-VPM pathway contributes to anxiety-like behaviors induced by malocclusion
Source: Front Cell Neurosci. 2022 Dec 20;16:995345. doi: 10.3389/fncel.2022.995345 (PMC9807610; doi:10.3389/fncel.2022.995345)

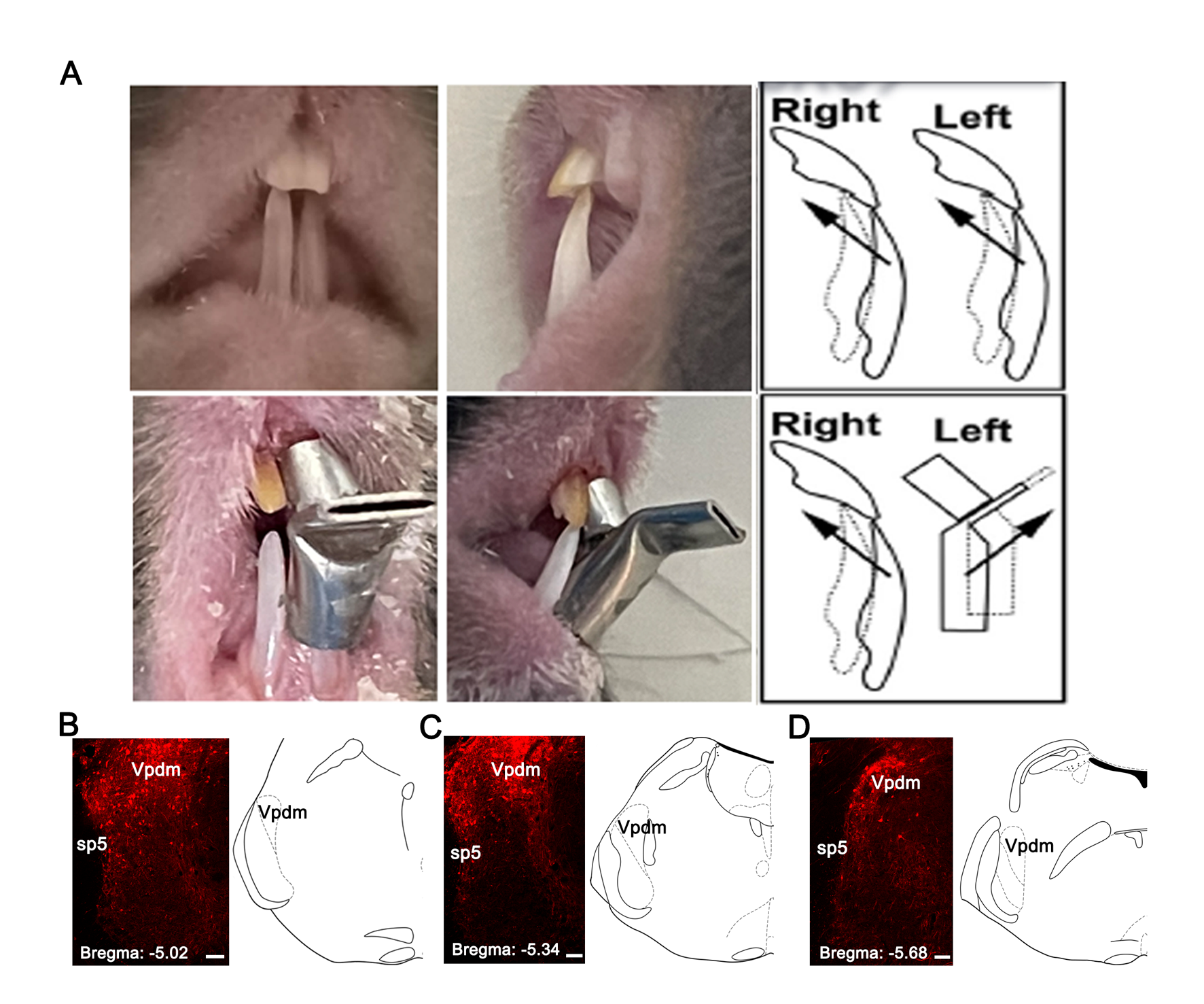

Supplement: Supplementary Figure 1 — (A) Schematic diagram and photograph of the UAC model. A section of metal tube with a length of 1.5 mm and an inner diameter of 0.3 mm was bonded onto the rat left maxillary incisor. A curved section of metal tube with a length of 2.5 mm and an inner diameter of 0.3 mm was adhered to the left mandibular incisor. The mandibular tube was bent and tilted to form a 135° angle with the occlusal plate. (B–D) Representative coronal section showing the rostrocaudal expression of the virus in the Vpdm (coordinates relative to Bregma: –5.02 mm, –5.34 mm and –5.68 mm). Scale bar: 200 μm. [file Image_1.tif]

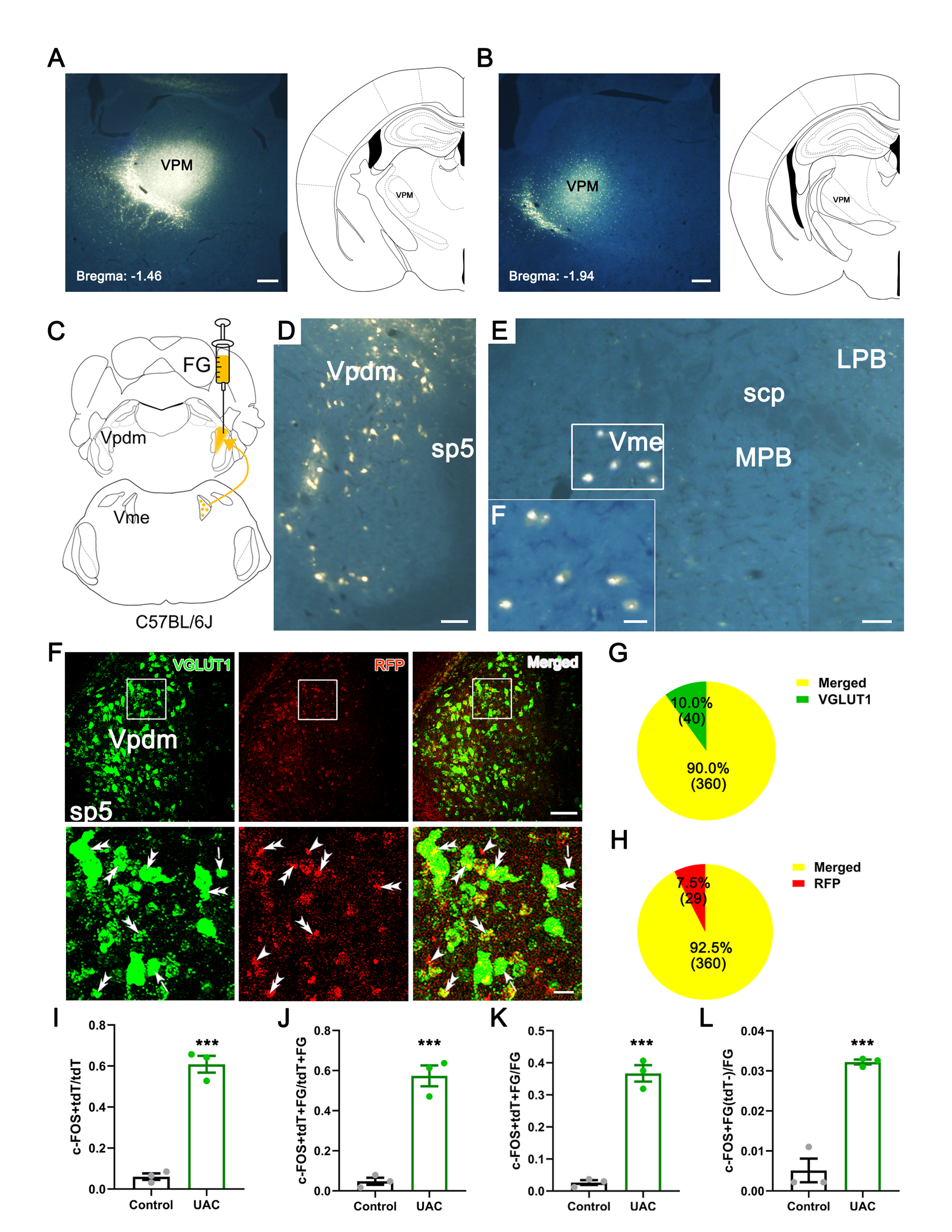

Supplement: Supplementary Figure 2 — (A,B) Representative coronal section showing the rostrocaudal expression of FG in the VPM (coordinates relative to Bregma –1.46 mm and –1.94 mm). Scale bar: 100 μm. (C,D) The Vme-Vpdm pathway was verified by injecting FG into the Vpdm. Scale bar: 200 μm. (E) FG-retrograde-labeled neuronal cell bodies were observed in the Vme. Scale bar: 100 μm. The enlarged FG-labeled neurons in the white square. Scale bar: 50 μm. (F) (Top) VGLUT1/RFP double-labeled neurons in the Vpdm in VGLUT1:tdTomato mice. Scale bar: 200 μm. (Bottom) The enlarged double-labeled neurons in the white square, with the double arrowheads indicating VGLUT1/RFP double-labeled neurons. The arrows indicate VGLUT1-labeled neurons. And the single arrowheads indicate the RFP -labeled neurons. Scale bar: 20 μm. (G,H) The pie chart shows the number of VGLUT1/RFP double-labeled neurons versus the total number of VGLUT1-labeled neurons and RFP-labeled neurons. (I,J) In VGLUT1:tdTomato mice, c-FOS/FG/tdTomato triple-stained neurons were observed in the 2-week control (n = 3) and UAC (n = 3) groups. (I) The activation of VpdmVGLUT1 neurons in UAC mice was displayed by the higher c-FOS + tdT/tdT ratio (the ratio of c-FOS/tdTomato double-stained neurons to tdTomato-labeled neurons). (J) The activation of the VGLUT1-ir neurons contained in the VpdmVGLUT1-VPM pathway in UAC mice was determined according to the higher c-FOS + tdT + FG/tdT + FG ratio (the ratio of c-FOS/tdTomato/FG triple-stained neurons to tdTomato/FG double-labeled neurons). (K) The activation of VGLUT1-ir neurons contained in the Vpdm-VPM pathway was displayed by the higher ratio of c-FOS + tdT + FG/FG in the UAC mice. (L) The activation of non-VGLUT1-ir neurons contained in the Vpdm-VPM pathway was determined according to the higher ratio of c-FOS + FG (tdT-)/FG in the UAC mice. Data are expressed as the mean ± SEM. ***P < 0.001. [file Image_2.tif]

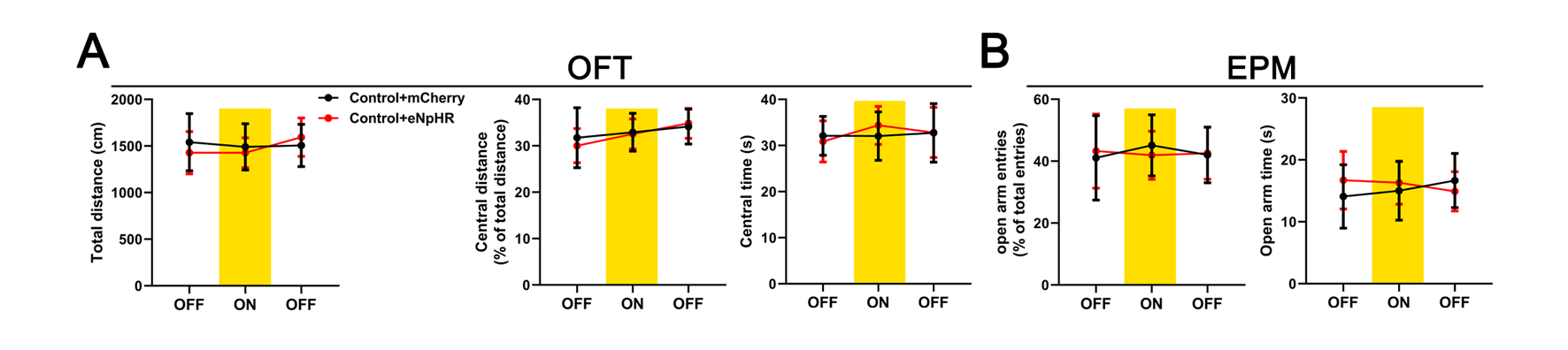

Supplement: Supplementary Figure 3 — Optogenetic inhibition of the VpdmVGLUT1-VPM pathway did not alter anxiety-like behaviors in the control group. (A,B) Optogenetic inhibition of the VpdmVGLUT1-VPM pathway did not affect locomotor abilities or anxiety-like behaviors in the OFT and EPM. Data are expressed as the mean ± SEM. Control + mCherry: n = 6; Control + eNpHR: n = 7. [file Image_3.tif]
