# Supplementary material for: Activation of the VpdmVGLUT1-VPM pathway contributes to anxiety-like behaviors induced by malocclusion
Source: Front Cell Neurosci. 2022 Dec 20;16:995345. doi: 10.3389/fncel.2022.995345 (PMC9807610; doi:10.3389/fncel.2022.995345)
Supplement: Supplementary file 4 [file Data_Sheet_1.docx]

**Table S1.** Data of behavioral tests in Figure 1 are given as P value of normality tests.

|  | OFT | | | | | | EPM | | | |
| --- | --- | --- | --- | --- | --- | --- | --- | --- | --- | --- |
|  | Total distance | | Central distance % | | Central time | | Open arm entries % | | Open arm time | |
|  | Con | UAC | Con | UAC | Con | UAC | Con | UAC | Con | UAC |
| 1 weeks | 0.5939 | 0.5749 | 0.8880 | 0.1900 | 0.1182 | 0.8317 | 0.1561 | 0.5546 | 0.2064 | 0.1869 |
| 2 weeks | 0.7613 | 0.3650 | 0.6841 | 0.7826 | 0.8146 | 0.4795 | 0.2997 | 0.2702 | 0.3413 | 0.3866 |
| 4 weeks | 0.1445 | 0.2572 | 0.6999 | 0.5824 | 0.5202 | 0.7124 | 0.2730 | 0.0810 | 0.2569 | 0.6028 |
| 6 weeks | 0.1824 | 0.7473 | 0.1004 | 0.5418 | 0.5702 | 0.6045 | 0.1799 | 0.4229 | 0.7106 | 0.5219 |

**Table S2.** Data of behavioral tests in Figure 2 are given as P value of normality tests.

|  | OFT | | | | | | EPM | | | |
| --- | --- | --- | --- | --- | --- | --- | --- | --- | --- | --- |
|  | Total distance | | Central distance % | | Central time | | Open arm entries % | | Open arm time | |
|  | Con | UAC | Con | UAC | Con | UAC | Con | UAC | Con | UAC |
| mCherry-CNO | 0.6236 | 0.9388 | 0.5933 | 0.8543 | 0.8229 | 0.2227 | 0.9119 | 0.6572 | 0.2292 | 0.8718 |
| hM4Di-SAL | 0.2748 | 0.9712 | 0.5544 | 0.8717 | 0.8231 | 0.1878 | 0.3542 | 0.5605 | 0.6772 | 0.8192 |
| hM4Di-CNO | 0.4672 | 0.4550 | 0.9701 | 0.3172 | 0.1993 | 0.8637 | 0.5052 | 0.7232 | 0.6899 | 0.4122 |

**Table S3.** Data of behavioral tests in Figure 3 are given as P value of normality tests.

|  | OFT | | | | | | EPM | | | |
| --- | --- | --- | --- | --- | --- | --- | --- | --- | --- | --- |
|  | Total distance | | Central distance % | | Central time | | Open arm entries % | | Open arm time | |
|  | Con | UAC | Con | UAC | Con | UAC | Con | UAC | Con | UAC |
| hM3Dq-SAL | 0.9679 | 0.2753 | 0.7037 | 0.5292 | 0.8813 | 0.3285 | 0.9804 | 0.6551 | 0.5935 | 0.3963 |
| hM3Dq-CNO | 0.5349 | 0.6536 | 0.6474 | 0.4279 | 0.6626 | 0.2274 | 0.9532 | 0.6094 | 0.6193 | 0.2288 |

**Table S4.** Data of behavioral tests in Figure 5 are given as P value of normality tests.

|  | OFT | | | | | | EPM | | | |
| --- | --- | --- | --- | --- | --- | --- | --- | --- | --- | --- |
|  | Total distance | | Central distance % | | Central time | | Open arm entries % | | Open arm time | |
|  | Con | UAC | Con | UAC | Con | UAC | Con | UAC | Con | UAC |
| mCherry-CNO | 0.3936 | 0.6889 | 0.9500 | 0.9956 | 0.1681 | 0.2552 | 0.1680 | 0.3704 | 0.7758 | 0.1798 |
| hM4Di-SAL | 0.9600 | 0.2107 | 0.6539 | 0.2217 | 0.7406 | 0.4425 | 0.1877 | 0.9470 | 0.6098 | 0.9730 |
| hM4Di-CNO | 0.9309 | 0.2424 | 0.9736 | 0.5200 | 0.9853 | 0.7381 | 0.7318 | 0.1526 | 0.3570 | 0.9793 |

**Table S5.** Data of behavioral tests in Figure 6 are given as P value of normality tests.

|  | OFT | | | | | | EPM | | | |
| --- | --- | --- | --- | --- | --- | --- | --- | --- | --- | --- |
|  | Total distance | | Central distance % | | Central time | | Open arm entries % | | Open arm time | |
|  | Con | UAC | Con | UAC | Con | UAC | Con | UAC | Con | UAC |
| hM3Dq-SAL | 0.1126 | 0.5226 | 0.9284 | 0.1784 | 0.1486 | 0.2193 | 0.1196 | 0.3684 | 0.2797 | 0.3767 |
| hM3Dq-CNO | 0.0851 | 0.0702 | 0.0512 | 0.4181 | 0.5975 | 0.1662 | 0.1226 | 0.2613 | 0.3133 | 0.0834 |

**Table S6.** Data of behavioral tests in Figure 7 and Supplementary Figure S3 are given as P value of normality tests.

|  | OFT | | | | | | EPM | | | |
| --- | --- | --- | --- | --- | --- | --- | --- | --- | --- | --- |
|  | Total distance | | Central distance % | | Central time | | Open arm entries % | | Open arm time | |
|  | Con | UAC | Con | UAC | Con | UAC | Con | UAC | Con | UAC |
| eNpHR-OFF1 | 0.4664 | 0.3208 | 0.4126 | 0.8399 | 0.3659 | 0.3105 | 0.1694 | 0.1894 | 0.1350 | 0.7716 |
| eNpHR-ON | 0.9845 | 0.8665 | 0.8728 | 0.5428 | 0.9257 | 0.9387 | 0.1417 | 0.0942 | 0.8025 | 0.7470 |
| eNpHR-OFF2 | 0.8995 | 0.5132 | 0.5271 | 0.0939 | 0.7221 | 0.7661 | 0.2411 | 0.5772 | 0.3573 | 0.5771 |
| mCherry-OFF1 | 0.4350 | 0.1523 | 0.2420 | 0.3880 | 0.0752 | 0.5820 | 0.1007 | 0.5633 | 0.5314 | 0.2240 |
| mCherry-ON | 0.1760 | 0.2756 | 0.7128 | 0.4846 | 0.6695 | 0.2787 | 0.1968 | 0.5596 | 0.6823 | 0.3554 |
| mCherry-OFF2 | 0.0508 | 0.0801 | 0.2162 | 0.1834 | 0.5289 | 0.1069 | 0.1008 | 0.3077 | 0.9322 | 0.6686 |
